# Biochemical and Pharmacological Characterization of the Human Lymphocyte Antigen B-Associated Transcript 5 (BAT5/ABHD16A)

**DOI:** 10.1371/journal.pone.0109869

**Published:** 2014-10-07

**Authors:** Juha R. Savinainen, Jayendra Z. Patel, Teija Parkkari, Dina Navia-Paldanius, Joona J. T. Marjamaa, Tuomo Laitinen, Tapio Nevalainen, Jarmo T. Laitinen

**Affiliations:** 1 School of Medicine, Institute of Biomedicine, Faculty of Health Sciences, University of Eastern Finland, Kuopio, Finland; 2 School of Pharmacy, Faculty of Health Sciences, University of Eastern Finland, Kuopio, Finland; Stanford University, United States of America

## Abstract

**Background:**

Human lymphocyte antigen B-associated transcript 5 (BAT5, also known as ABHD16A) is a poorly characterized 63 kDa protein belonging to the α/β-hydrolase domain (ABHD) containing family of metabolic serine hydrolases. Its natural substrates and biochemical properties are unknown.

**Methodology/Principal Findings:**

Amino acid sequence comparison between seven mammalian BAT5 orthologs revealed that the overall primary structure was highly (≥95%) conserved. Activity-based protein profiling (ABPP) confirmed successful generation of catalytically active human (h) and mouse (m) BAT5 in HEK293 cells, enabling further biochemical characterization. A sensitive fluorescent glycerol assay reported hBAT5-mediated hydrolysis of medium-chain saturated (C14∶0), long-chain unsaturated (C18∶1, C18∶2, C20∶4) monoacylglycerols (MAGs) and 15-deoxy-Δ^12,14^-prostaglandin J_2_-2-glycerol ester (15d-PGJ_2_-G). In contrast, hBAT5 possessed only marginal diacylglycerol (DAG), triacylglycerol (TAG), or lysophospholipase activity. The best MAG substrates were 1-linoleylglycerol (1-LG) and 15d-PGJ_2_-G, both exhibiting low-micromolar K_m_ values. BAT5 had a neutral pH optimum and showed preference for the 1(3)- vs. 2-isomers of MAGs C18∶1, C18∶2 and C20∶4. Inhibitor profiling revealed that β-lactone-based lipase inhibitors were nanomolar inhibitors of hBAT5 activity (palmostatin B > tetrahydrolipstatin > ebelactone A). Moreover, the hormone-sensitive lipase inhibitor C7600 (5-methoxy-3-(4-phenoxyphenyl)-3H-[1], [3], [4]oxadiazol-2-one) was identified as a highly potent inhibitor (IC_50_ 8.3 nM). Phenyl and benzyl substituted analogs of C7600 with increased BAT5 selectivity were synthesized and a preliminary SAR analysis was conducted to obtain initial insights into the active site dimensions.

**Conclusions/Significance:**

This study provides an initial characterization of BAT5 activity, unveiling the biochemical and pharmacological properties with *in vitro* substrate preferences and inhibitor profiles. Utilization of glycerolipid substrates and sensitivity to lipase inhibitors suggest that BAT5 is a genuine lipase with preference for long-chain unsaturated MAGs and could in this capacity regulate glycerolipid metabolism *in vivo* as well. This preliminary SAR data should pave the way towards increasingly potent and BAT5-selective inhibitors.

## Introduction

The human serine hydrolases form a large family of enzymes with a predicted number of ∼240 that fall into two subfamilies: the serine proteases (∼125 members) and the metabolic serine hydrolases (∼115 members) [1], [2]. The metabolic serine hydrolases include small-molecule hydrolases, such as lipases, esterases and amidases and utilize a conserved serine nucleophile to hydrolyze e.g. amide, ester, and thioester bonds. The metabolic serine hydrolases are often characterized by a α/β-hydrolase domain (ABHD) fold and typically use a Ser-His-Asp (SHD) triad for catalysis. Although many of these hydrolases are well known, several remain poorly characterized with respect to their substrate preferences, inhibitor profiles and physiological functions [3].

BAT5 (human lymphocyte antigen B-associated transcript 5, also known as ABHD16A) remains an unannotated 63 kDa (558 amino acid residues) protein classified to the ABHD family of metabolic serine hydrolases [3]–[5]. The biochemical function, substrates, and products of BAT5 activity have not been identified. BAT5 belongs to a cluster of genes within the human major histocompatibility complex (MHC) class III, indicating that BAT5 may regulate immunity [6]–[7]. In humans, BAT5 polymorphism has been associated with susceptibility to Kawasaki disease and coronary artery aneurysm [8]. In pigs, a single nucleotide polymorphism in BAT5 was found to associate with back fat thickness [9], suggesting that BAT5 might be involved in adipose tissue function and lipid metabolism. BAT5 is predicted to be an integral membrane protein with highest mRNA transcript levels in mouse tissues found in testis, heart, muscle, and brain [3].

Although no substrate-based activity assays have been described to date, BAT5 activity can be readily detected in native proteomes using the chemoproteomic approach known as activity-based protein profiling (ABPP) with the active site serine-directed fluorophosphonate (FP) probes [4], [5]. A previous study has indicated that in addition to the broadly acting lipase inhibitor methylarachidonoyl fluorophosphonate (MAFP), the β-lactone tetrahydrolipstatin (THL, also known as orlistat) dose-dependently prevented the FP probe binding to this serine hydrolase in native brain membrane proteomes and lysates of HEK293 cells overexpressing hBAT5 [4].

We have devised a sensitive methodology allowing kinetic detection of glycerol formed in the hydrolysis of MAGs, catalyzed by the serine hydrolases ABHD6, ABHD12 and MAG lipase (MAGL) [10]. This methodology has facilitated the substrate and inhibitor profiling of these hydrolases, allowing parallel testing of a variety of natural MAGs, as well as additional glycerolipid substrates such as prostaglandin glycerol esters (PG-Gs) [10]–[11]. Here we have adopted this methodology in combination with ABPP in an effort to unveil the substrate preferences and inhibitor profiles of BAT5. We show that after transient expression in HEK293 cells, human BAT5 (hBAT5) catalyzed the hydrolysis of a restricted set of MAGs and PG-Gs, most notably 1-linoleylglycerol (1-LG) and 15-deoxy-Δ^12,14^-prostaglandin J_2_-2-glycerol ester (15d-PGJ_2_-G). In contrast, hBAT5 did not utilize DAGs or TAGs. Furthermore, hBAT5 exhibited no detectable lysophospholipase activity towards lysophosphatidic acid (LPA) or lysophosphatidyl serine (LPS). Inhibitor profiling revealed that hBAT5 was sensitive to various lipase inhibitors, including the β-lactones palmostatin B, THL and ebelactone A. Moreover, the hormone-sensitive lipase inhibitor C7600 was identified as a highly potent hBAT5 inhibitor (IC_50_ 8.3 nM). Structural modifications of the 1,3,4-oxadiazol-2(3H)-one backbone of C7600 yielded compounds with improved BAT5 selectivity and a preliminary SAR analysis based on these compounds was conducted to obtain initial insights into the active site. Our *in vitro* study suggests that BAT5 is a genuine MAG lipase with preference for long-chain unsaturated MAGs and could in this capacity regulate glycerolipid metabolism *in vivo* as well.

## Results and Discussion

### The primary structure of mammalian BAT5 is highly conserved

**Figure 1:**
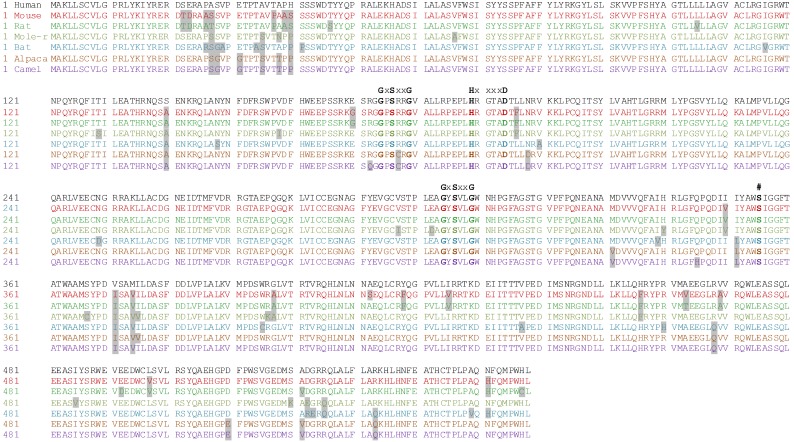
Comparisons of primary structures between mammalian BAT5 orthologs. Predicted acyltransferase motif (**H**xxxx**D**) [3] and predicted active site nucleophile (**#**) [3] are idicated. In addition, two lipase-like motifs (**G**x**S**xx**G**) are highlighted. Gray shading indicates amino acid residues deviating from the human sequence. Comparison to the human sequence indicated the following identity: mouse (96%), rat (95%), naked mole rat (96%), bat (95%), alpaca (97%) and camel (97%). The following gene bank accession numbers were used for the sequences: Human (*Homo sapiens*) BAB63383.1, mouse (*Mus muculus*) NP_848707.1, rat (*Rattus norvegicus*) NP_997696.1, naked mole-rat (*Heterocephalus glaber*) XP_004847034, bat (*Myotis lucifugus*) XP_006104671, alpaca (*Vicugna pacos*) XP_006215394, camel (*Camelus ferus*) XP_006178831.

As an initial step in the characterization of BAT5, we compared the primary structures of the full-length (558 amino acids) proteins between human, rodent and more exotic mammalian species, including the naked mole-rat which has an extraordinary longevity and cancer resistance [12] (Figure 1). This comparative analysis revealed that the overall primary structure of the BAT5 orthologs was highly conserved between human and mouse (96%), rat (95%), naked mole rat (96%), bat (95%), alpaca (97%), and camel (97%). The two predicted motifs [3], namely active site nucleophile (S355) and acyltransferase motif (HxxxxD), were fully conserved. In addition, sequence comparisons indicated the presence of two fully conserved and identical lipase-like motifs (GxSxxG instead of the canonical GxSxG lipase motif). The high degree of evolutionary conservation suggests that BAT5 likely evolved to mediate closely related functions in mammalian species as divergent as human, bat and camel.

### HEK293 cells as a convenient host for transient expression of catalytically active BAT5

**Figure 2:**
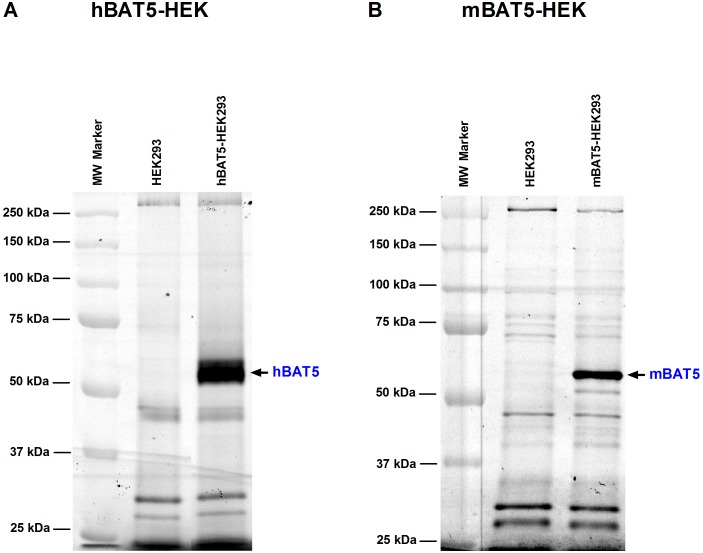
Activity-based protein profiling (ABPP) to visualize catalytically active BAT5 protein in lysates of HEK293 cells after transient expression of human (h) or mouse (m) BAT5 orthologs. Serine hydrolases were labeled using the active site serine targeting fluorescent probe TAMRA-FP. After separation in SDS-electrophoresis gel (10%), serine hydrolase activity was visualized by in-gel fluorescent gel scanning as detailed in the Methods section. Molecular weight markers (MW) are indicated at left. Transient transfections with the cDNAs encoding hBAT5 (**A**) or mBAT5 (**B**) results in robust labeling of a ∼63 kDa protein band that is absent from parental HEK293 cells. Data are from one typical transfection, transfections were repeated twice with similar outcome.

Our previous study has indicated that HEK293 cells serve a convenient host for transient expression of the ABHD family members ABHD6 and ABHD12 [10]. We chose HEK293 cells as the host for the expression of BAT5 as well. Cell lysates were prepared 48 h after transient transfections with the cDNAs encoding hBAT5 or mBAT5. ABPP confirmed that the BAT5 orthologs were expressed as catalytically active proteins with the expected (63 kDa) size (Figure 2). The ABPP also indicated that HEK293 cells express negligible endogenous BAT5 activity, as there was no visible TAMRA-FP labeling of endogenous serine hydrolases migrating at the 63 kDa size-range in parental cells.

### BAT5 is a MAG lipase with preference for 1-LG and 15d-PGJ_2_-G

**Figure 3:**
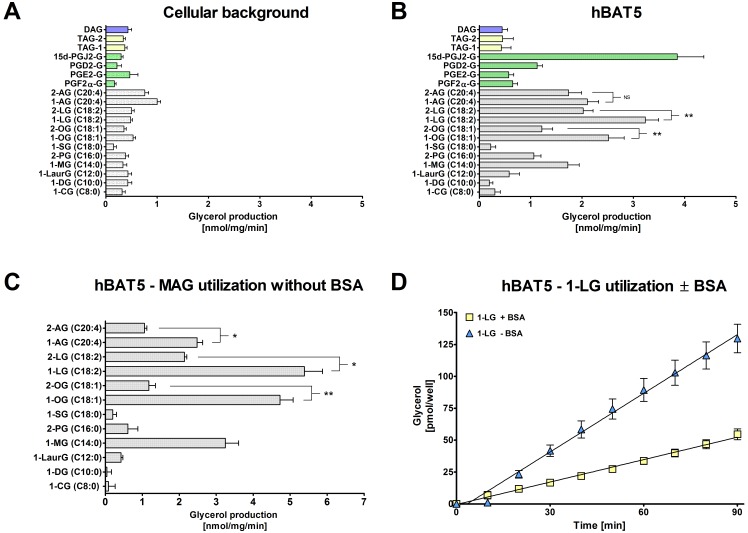
*In vitro* glycerolipid substrate profile of hBAT5. HEK293 cells were transiently transfected with the cDNA encoding hBAT5, as detailed in the Methods section. After 48 h, cells were harvested and lysates prepared for hydrolase activity measurements using a sensitive fluorescent glycerol assay. The substrate panel included monoacylglycerols (MAGs) with the indicated acyl chain length, isomer and degree of saturation, the diacylglycerol (DAG) 1,2-dioleoyl(C18∶1)-*rac-*glycerol, the triacylglycerols (TAG-1 = 1,2,3-trioleoyl(C18∶1)glycerol; TAG-2 = 1-palmitoyl(C16∶0)-2-oleoyl(C18∶1)-3-linoleoyl(C18∶2)-*rac*-glycerol, as well as the prostaglandin glycerol esters PGD_2_-G, PGE_2_-G, PGF_2α_-G and 15d-PGJ_2_-G. Cellular lysates (0.3 µg/well) were incubated together with the indicated substrates [25 µM final concentration, added from 10 mM stock solutions in ethanol into the glycerol assay mix containing 0.5% (w/v) fatty acid free BSA and 1% (v/v ethanol). Glycerol production was determined at time-point 60 min. **A.** Background activity for the tested substrates. Cellular background was similar between HEK and Mock-transfected cells and the values shown are combined from the two. **B.** Substrate profile of hBAT5. **C.** The MAG substrate profile of hBAT5 in assays conducted without BSA. **D.** Linear, time-dependent generation of glycerol as a result of 1-LG (25 µM) hydrolysis in hBAT5-HEK lysates (0.3 µg/well) in incubations with or without BSA. Note ∼2.5-fold higher hydrolysis rate in the absence of BSA. Data are mean + SEM from 3–7 (A and B) or three (C and D) independent experiments using hBAT5 lysates from one transfection. Statistical comparisons between the MAG 1(3)- and 2-isomers were done by using unpaired (B) or paired (C) t-test and the significance is indicated with an asterix (NS, non-significant; *, p<0.05; **, p<0.01).

We have previously shown that the fluorescent glycerol assay offers low picomolar sensitivity and enables MAG hydrolase activity measurements using merely 0.3 µg lysate protein per well, ensuring negligible background activity [10]. Here, we tested the substrate preferences of BAT5 using lysates of hBAT5-HEK293 cells in assay mixes containing MAGs, DAGs and TAGs with varying acyl chain length and saturation, as detailed in Figure 3. The substrates were tested at 25 µM final concentration and BSA [0.5% (w/v), essentially fatty acid free] was included as a carrier for the lipophilic compounds.

Background activity was similar for all tested substrates between parental and Mock-transfected cells (Figure S1), and the mean values were chosen to represent the cellular background activity (Figure 3A). As illustrated in Figure 3B, hBAT5 hydrolyzed the tested MAGs with a distinct substrate preference. MAGs with saturated acyl chain lengths between C8-C12 were relatively poor substrates, as was also MAG C18∶0. On the other hand, MAGs C14∶0, C16∶0, C18∶1, C18∶2 and C20∶4 were hydrolyzed, albeit with variable speed. The relative rate order was C18∶2 > C18∶1 > C20∶1 ≈ C14∶0 > C16∶0. The preference for 1(3)- vs. 2-MAG isomers was tested using oleoyl (C18∶1), linoleoyl (C18∶2) and arachidonoyl (C20∶4) glycerols and it was found that hBAT5 hydrolyzed significantly faster the 1(3)-isomers of C18∶1 and C18∶2 whereas there was no statistically significant difference in the hydrolysis rates between the C20∶4 isomers.

In addition to the MAGs, we assessed the capacity of hBAT5 to hydrolyze prostaglandin glycerol esters (PG-Gs), the products of cyclooxygenase-2 catalyzed 2-arachidonoylglycerol (2-AG) oxygenation [13]–[14]. The inclusion of PG-Gs was of interest in light of recent *in vitro* findings showing that the PG-Gs were good substrates for the serine hydrolases MAGL [11], carboxylesterase 1 (CES1) and palmitoyl protein thioesterase 1 (PPT1) [15]–[16]. From the tested PG-Gs, hBAT5 readily hydrolyzed 15d-PGJ_2_-G and to lesser extend also prostaglandin D_2_-1-glycerol ester (PGD_2_-G) (Figure 3B). In contrast, hydrolysis rates of prostaglandin E_2_- and F_2α_-1-glycerol esters (PGE_2_-G and PG-F_2α_-G, respectively) were low. hBAT5 showed only marginal activity towards the tested DAG and TAG species (Figure 3B).

As our hydrolase assays routinely contain 0.5% BSA as a carrier for lipophilic compounds, we were curious to find out whether the BAT5 substrate preference would be similar if tested in the absence of BSA. These experiments indicated that the overall MAG substrate profile was grossly similar regardless of whether BSA was included or not (Figure 3B–C). However, a notable difference was that barely detectable hydrolysis of MAGs C16∶0 took place in the absence of BSA. In addition, the preference for the 1(3)-isomers over the 2-isomers of C18∶1, C18∶2 and C20∶4 was more pronounced in incubations without BSA, suggesting that the availability of the 1(3)-isomers for hydrolysis was facilitated (or that of the 2-isomers diminished) in the BSA-free environment.

From the MAG series, 1-LG was the best substrate and the kinetic assay confirmed that hBAT5-catalyzed 1-LG hydrolysis generated glycerol in a linear manner during the 90 min incubation period under both conditions (Figure 3D). It is noteworthy that the rate of 1-LG hydrolysis was ∼2.5-fold higher in assays without BSA (Figure 3D). Due to financial limitations, BAT5 utilization of PG-Gs was tested only using the routine protocol with BSA but our preliminary experiments indicated that the availability of 15d-PGJ_2_-G was not drastically different between assays with or without BSA (unpublished data). Collectively these results demonstrate that inclusion of BSA is justified as a carrier for lipophilic substrates, as exemplified for MAG C16∶0 but that for particular substrates, like MAG C18∶2 (1-LG), the carrier may avidly bind the substrate, thereby limiting substrate availability for the enzyme. Importantly, however, the BAT5 substrate profile was comparable under both conditions.

### BAT5 shows undetectable lysophospholipase activity

**Figure 4:**
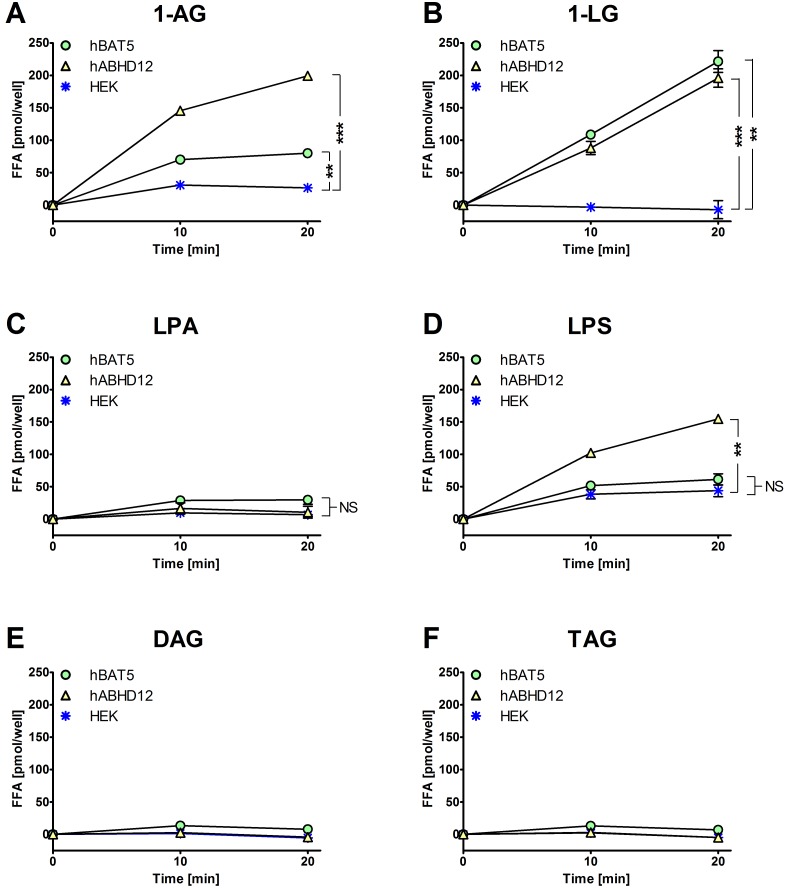
Fatty acid liberation from various substrates when incubated together with lysates of HEK293 cells expressing hBAT5 or hABHD12. HEK293 cells were transiently transfected with the cDNA encoding hBAT5 or hABHD12, as detailed in [10] and the Methods section. After 48 h, cells were harvested and lysates prepared for lipase activity measurements based on the Cayman’s FFA fluorescence assay kit. For validation purposes, lysates of parental HEK293 cells were tested in parallel, and the substrate panel included the MAGs 1-AG (**A**) and 1-LG (**B**), the lysophospholipids C18∶1-LPA (C) and C18∶1-LPS (D), the DAG 1,2-dioleoyl(C18∶1)-*rac-*glycerol (E), and the TAG 1,2,3-trioleoyl(C18∶1)glycerol (F). Cellular lysates (0.3 µg/well) were incubated together with the indicated substrates [25 µM final concentration, added from 10 mM stock solutions in ethanol into the FFA assay mix containing 0.1% (v/v ethanol). Assay blank and fatty acid standard (C18∶1) were included for each condition. FFA content was determined at time-points 10 and 20 min. Data are mean ± SEM from quadruplicate wells in one experiment. Statistical comparisons to values of the HEK lysates were done using paired t-test and the significance is indicated with an asterix (NS, non-significant; **, p<0.01; ***, p<0.001).

We evaluated lysophospholipase activity using 1-oleoyl-lysophosphatidic acid (C18∶1-LPA) and 1-oleoyl-lysophosphatidylserine (C18∶1-LPS) as hBAT5 substrates, and used free fatty acid (FFA) release in these assays as the readout for lipase activity. A commercially available FFA kit was tuned to closely mimic the glycerol assay. Therefore, we first validated the method by testing the ability of this assay to detect hydrolysis of selected MAG substrates by hBAT5 and hABHD12 (as a positive control). FFA release was tested in the absence of BSA. We found that in agreement with the outcome of the glycerol assay (Ref. [10] and Figure 3 of the present study), hBAT5 and hABHD12 hydrolyzed the MAG substrates 1-AG and 1-LG in a time-dependent manner (Figure 4A–B). In contrast, no statistically significant liberation of oleic acid (C18∶1) exceeding the cellular background was found for hBAT5 with LPA or LPS as the substrates (both tested at 25 µM final concentration) (Figure 4C–D). In line with previous results [10], [17], hABHD12 catalyzed oleic acid release from LPS but not from LPA (Figure 4C–D). Finally, neither hBAT5 nor hABHD12 catalyzed oleic acid release from the tested DAG or TAG species (Figure 4E–F), as expected based on the results of the glycerol assay (Ref. [10] and Figure 3 of the present study). The outcome was essentially the same when 1-AG, 1-LG, LPS and TAG was tested at 50 µM final concentration (Figure S2).

It could be argued that the observed substrate profile does not necessarily reflect true hBAT5 preference but rather availability of the lipophilic substrates for the enzyme. However, several lines of evidence indicate that this is not the case. Our previous study [10] has demonstrated that the MAGs C8∶0, C10∶0 and C12∶0 (all poorly utilized by hBAT5) were efficiently hydrolyzed under identical conditions by the serine hydrolases hABHD6 and hMAGL, indicating that the substrates were readily available. We calculated the logP values for the twelve MAG species tested in the present study and plotted hBAT5 substrate utilization against the logP values (Figure S2). This analysis indicated that the best substrates were also highly lipophilic. It is noteworthy that 1-SG (C18∶0), representing the most lipophilic compound in this series (Figure S2), was not utilized by hBAT5 (Fig. 3B) but was modestly hydrolyzed by hMAGL under identical conditions [10]. Thus we believe that the presently used methodology has accurately revealed hBAT5 substrate preferences. Collectively, the substrate profiling studies demonstrated that hBAT5 acts as a genuine MAG lipase with preference towards the MAG species C14∶0, C16∶0, C18∶1, C18∶2 and C20∶4, as well as towards one PG-G, namely15d-PGJ_2_-G. Under the assay conditions employed, hBAT5 showed only marginal activity towards DAGs, TAGs or the lysophospholipids LPA and LPS.

### BAT5 has a pH optimum at the neutral range

**Figure 5:**
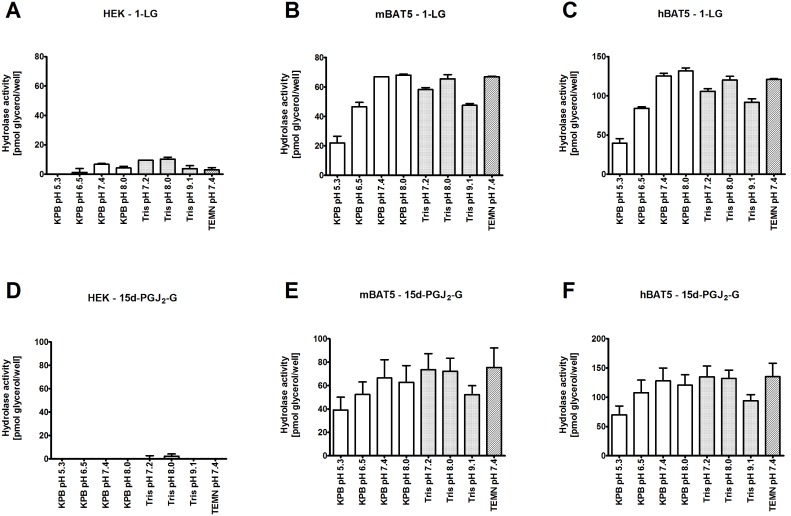
pH optima of mouse (m) and human (h) BAT5 hydrolase activities towards 1-LG and 15d-PGJ_2_-G. Lysates (0.3 µg/well) of HEK293 cells transiently expressing mBAT5 (**B**, **E**) or hBAT5 (**C**, **F**) were incubated at the indicated pH for 60 min using 1-LG (**A**, **B**, **C**) or 15d-PGJ_2_-G (**D**, **E**, **F**) as the substrate (25 µM final concentration). For comparative purposes, substrate hydrolysis in lysates of parental HEK293 cells is also illustrated (**A**, **D**). The incubations additionally contained 0.1% (v/v) ethanol, 0.1% (v/v) DMSO and 0.5% (w/v) BSA. After 60 min incubation, the pH of the assay system was brought to the neutral range by the addition of Glycerol Assay Mix containing additionally 100 mM Tris-HCl, pH 7.4 and THL (10^−5^ M) to quench hydrolase activity. Assay blanks and glycerol standards were included for each pH condition. Glycerol content was determined at time-point 60 min. The buffer systems were as follows: 10 mM K-phosphate buffer (KPB) covering the pH range 5.3–8.0 and 10 mM Tris-HCl covering the pH range 7.2–9.1. For comparative purposes, hydrolase activity in the routinely used hydrolase assay buffer (TEMN, pH 7.4) is shown. Data are mean + SEM of duplicate wells from two independent experiments.

The pH dependence of BAT5-catalyzed hydrolysis of the preferred substrates (1-LG and 15d-PGJ_2_-G) was evaluated in different buffer systems covering the pH range from 5.3 to 9.1 (Figure 5). These studies indicated that hBAT5 and mBAT5 optimally hydrolyzed both substrates in the pH range between 7.2 and 8.0. As the TEMN-BSA buffer (pH 7.4) has been extensively used in our previous studies characterizing serine hydrolase activity in HEK293 cell lysates [10]–[11], we also used this buffer (mainly with BSA but for particular cases also without BSA, as detailed in the text) in the present study.

### K_m_ and V_max_ values for 1-LG and 15d-PGJ_2_-G

**Figure 6:**
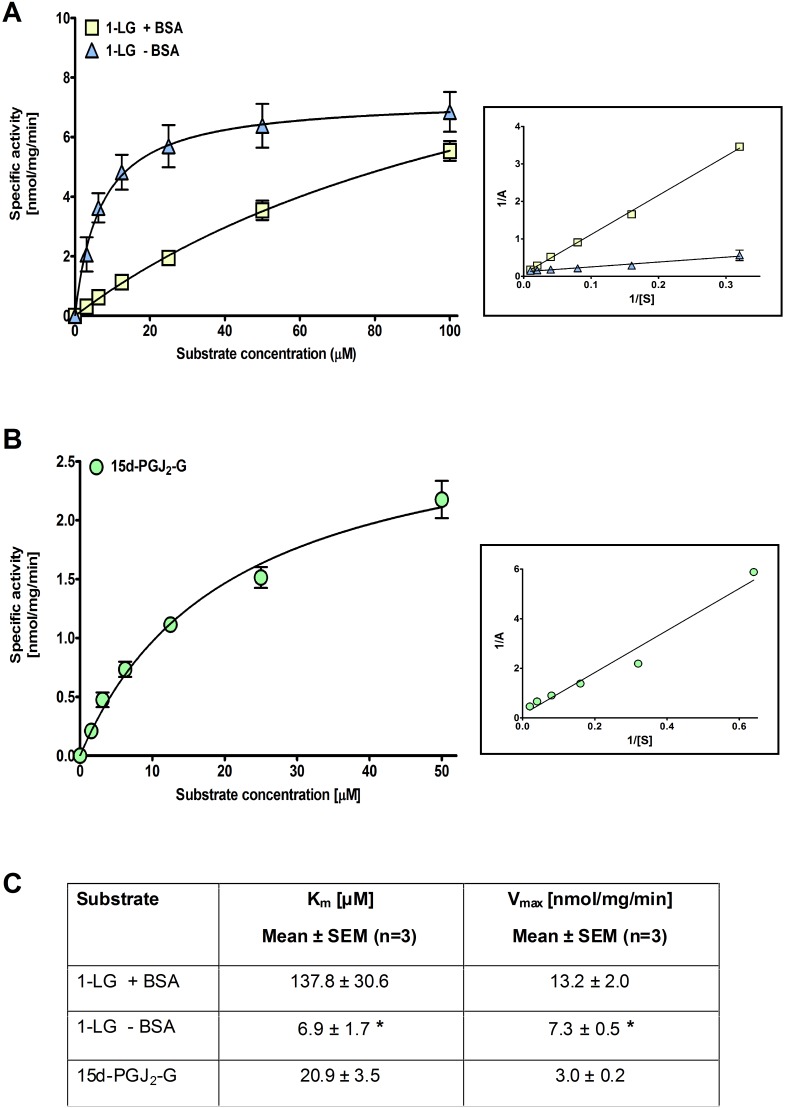
K_m_ and V_max_ values for hBAT5-dependent hydrolysis of 1-LG and 15d-PGJ_2_-G. HEK293 cells were transiently transfected with the cDNA encoding hBAT5, as detailed in the Methods section. After 48 h, cells were harvested and lysates prepared for hydrolase activity measurements. Cellular lysates (0.3 µg/well) were incubated together with the indicated concentrations of 1-LG in assay mixes containing (+ BSA) or not (– BSA) 0.5% (w/v) BSA (**A**) or with 15d-PGJ_2_-G in assay mix containing BSA (**B**). The K_m_ and V_max_ values (**C**) were determined at time-point 90 min, where substrate utilization was <10%, and were calculated as non-linear regressions using GraphPad Prism 5.0 for Windows. As crude cellular lysates were used, total protein concentration was used instead of true enzyme concentration. The Lineweaver-Burk plots are shown inside rectangles. Statistical comparison of the K_m_ and V_max_ values for 1-LG between the + and - BSA conditions was done using unpaired t-test and the significance is indicated with an asterix (*, p<0.05). Values are mean ± SEM from three independent experiments.

Using Michaelis-Menten analysis, we determined the K_m_ and V_max_ values for hBAT5-catalyzed hydrolysis of 1-LG in the absence and presence of BSA, and that of 15d-PGJ_2_-G in the presence of BSA. The tested substrate concentration range was 0–100 µM for 1-LG and 0–50 µM for 15d-PGJ_2_-G (Figure 6). For 1-LG, the K_m_ values were 137.8±30.6 and 6.9±1.7 µM in assays with and without BSA, respectively. The difference between the K_m_ values was statistically significant (p<0.05). For 15d-PGJ_2_-G, the K_m_ value was 20.9±3.5 µM. The maximal rates (V_max_) of 1-LG hydrolysis were 13.2±2.0 and 7.3±0.5 nmol/mg/min in assays with and without BSA, respectively. The difference between the V_max_ values was statistically significant (p<0.05). For 15d-PGJ_2_-G, the V_max_ value was 3.0±0.2 nmol/mg/min.

We are not aware of any prior published data regarding BAT5 substrate characterization, which precludes direct comparison. However, the K_m_ value (∼140 µM) obtained here for 1-LG in the presence of BSA is similar to the K_m_ values (110–160 µM) previously reported for hABHD6, hABHD12 and hMAGL under comparable assay conditions with 2-AG as the substrate [10]. Notably, the K_m_ value of 1-LG in assays without BSA was significantly (∼20-fold) smaller compared to the value in assays containing BSA, indicating that the carrier avidly bound this substrate, limiting its availability for enzymatic hydrolysis. We reported previously that 15d-PGJ_2_-G was a good substrate for hMAGL *in vitro* [11]. The K_m_ value (∼15 µM) determined in that study is comparable to the K_m_ value (∼21 µM) determined here for hBAT5 with this substrate. As discussed earlier [10], and further illustrated here for 1-LG in particular, the K_m_ values for lipophilic substrates should be considered only as approximates because of several factors that may affect the accuracy of K_m_ determinations. Factors, including availability of the lipophilic substrate due to limited solubility, tight binding to protein carriers, and/or critical micellar concentration, could all compromise accurate K_m_ determinations. Despite these limitations, such studies offer a comparative view to the ABHD family of serine hydrolases, as the K_m_ values have been determined under similar conditions.

### BAT5-mediated 1-LG hydrolysis is potently inhibited by the β-lactone palmostatin B

**Figure 7:**
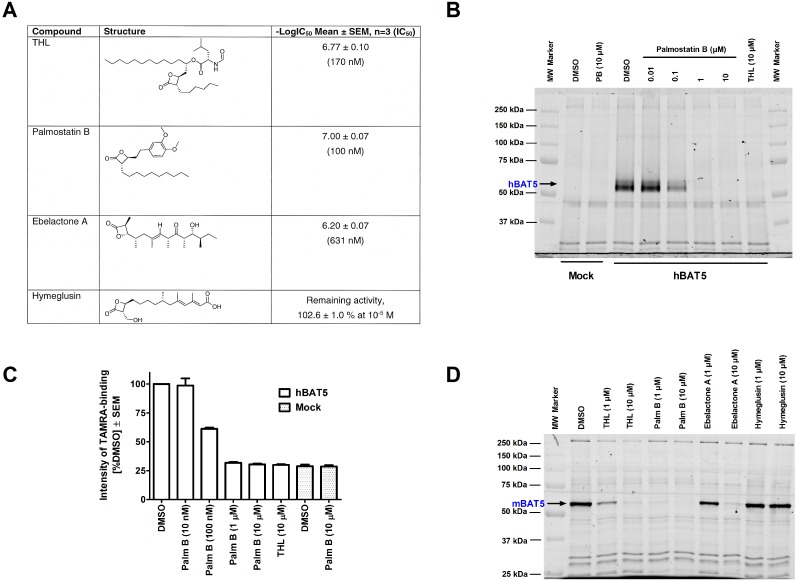
β-lactones as BAT5 inhibitors. **A.** Potency of β-lactones to inhibit 1-LG hydrolysis in lysates of hBAT5-HEK293 cells. Lysates were treated for 30 min with the inhibitors after which glycerol generated in the hydrolysis of 1-LG (25 µM final concentration) was monitored for 90 min. Inhibitor dose–response curves and IC_50_ values derived thereof were calculated from non-linear regressions using GraphPad Prism 5.0 for Windows. The logIC_50_ values are means ± SEM from three independent experiments. IC_50_ values (mean) are shown in parenthesis. **B–C.** Competitive ABPP of hBAT5-HEK293 cell lysates showing that palmostatin B inhibits probe binding to hBAT5 with the same potency (IC_50_ ∼ 100 nM) as it blocks hBAT5-dependent 1-LG hydrolysis (**A**). **C.** Quantitative data (mean + SEM) on the effects of palmostatin B and THL on probe labeling of the hBAT5 band (black arrow in B). The data are derived from three separate ABPP experiments. **D.** Competitive ABPP demonstrating that the potency order of the β-lactones to inhibit probe binding to mBAT5 (palmostatin B > THL > ebelactone A << hymeglusin (inactive) is identical to that observed for hBAT5 (**A**). In B and D, lysates (25 µg) were pretreated for 1 h with DMSO or the indicated concentrations of the inhibitors, after which TAMRA-FP labelling was conducted for 1 hour at RT. The reaction was stopped, 5 µg protein was loaded per lane and the proteins separated in SDS-electrophoresis minigel (10%). TAMRA-FP labeling was visualized after in-gel fluorescence imaging as described in the Methods section. The image in D is representative from two ABPP experiments with similar outcome.

THL was previously reported to inhibit BAT5 activity in competitive ABPP experiments with native mouse and recombinant human enzyme [4], [18]. We evaluated the potency of THL and additional compounds with the β-lactone scaffold (palmostatin B, ebelactone A and hymeglusin) as inhibitors of 1-LG hydrolysis in hBAT5-HEK293 lysates. Dose-response curves were constructed for each inhibitor using 5–6 concentrations and the IC_50_-values were calculated after nonlinear fitting of these curves. These studies indicated that the β-lactones inhibited hBAT5 activity with the relative potency order: palmostatin B (IC_50_ 100 nM) > THL (IC_50_ 170 nM) > ebelactone A (IC_50_ 630 nM) >> hymeglusin (inactive) (Figure 7A). As a complementary approach, competitive ABPP with hBAT5-HEK293 lysates was performed and these studies indicated that the potency of palmostatin B to inhibit probe binding to hBAT5 (IC_50_ ∼100 nM) was practically identical with the value obtained in the glycerol assay (Figure 7B–C). Competitive ABPP with mBAT5-HEK293 lysates demonstrated further that the relative potency of the β-lactones towards the mouse ortholog was identical to that observed for the human enzyme (Figure 7D).

**Figure 8:**
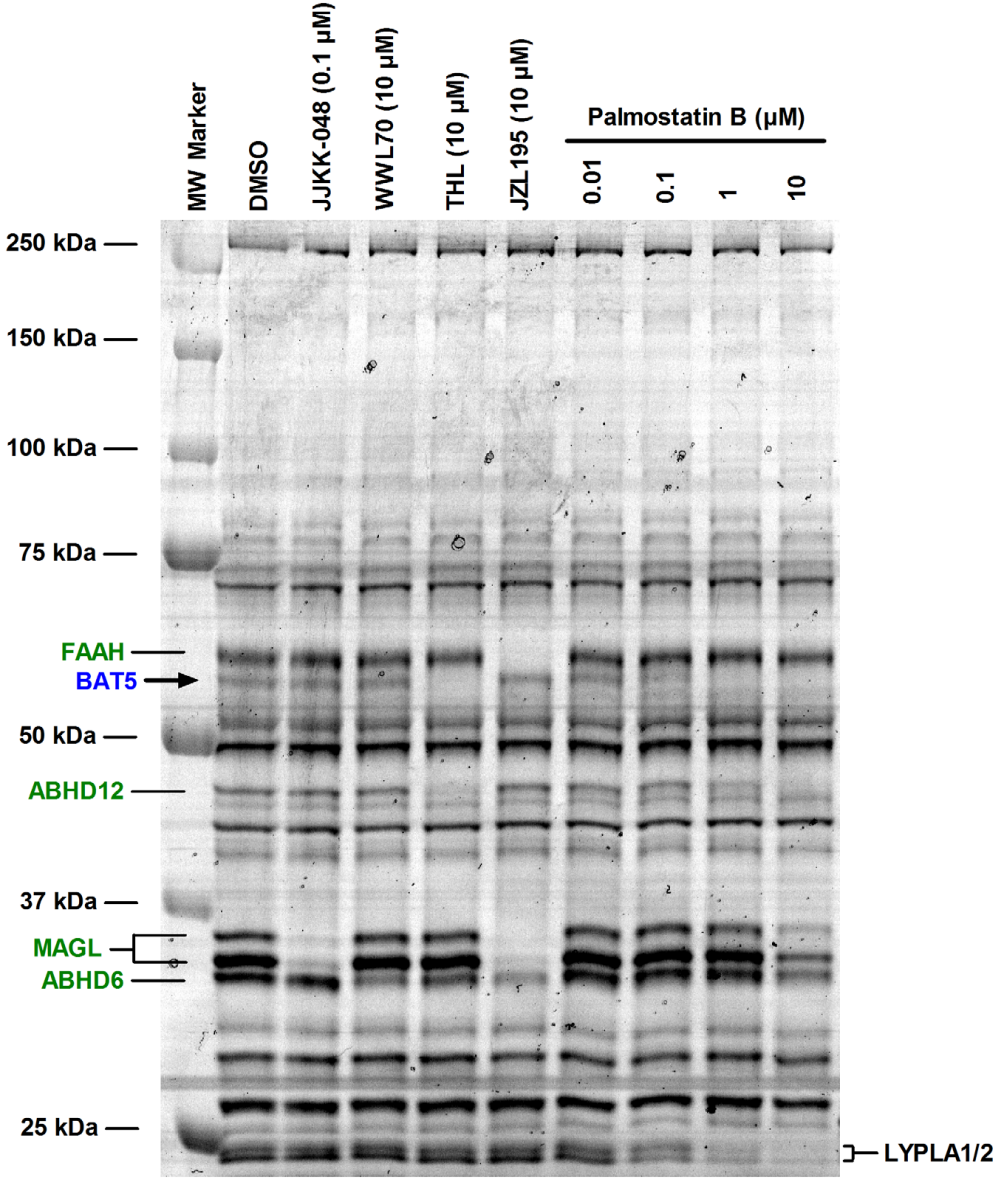
Competitive ABPP unveiling palmostatin B targets among the serine hydrolases in mouse brain membrane proteome. Membranes (100 µg) were pretreated for 1 h with DMSO or the indicated concentrations of the serine hydrolase inhibitors, after which TAMRA-FP labelling was conducted for 1 hour at RT. The reaction was stopped, 10 µg protein (10 µl) was loaded per lane and the proteins were separated in SDS-electrophoresis gel (10%). TAMRA-FP labeling was visualized after in-gel fluorescence imaging as described in the Methods section. Molecular weight markers are indicated at left. Reference inhibitors JJKK-048, WWL70, THL and JZL195 were used at the indicated concentrations to identify the following serine hydrolases from the gel: fatty acid amide hydrolase (FAAH), inhibited by JZL195 [21]; MAGL doublet, inhibited by JJKK-048 [22] and JZL195 [21]; ABHD6, inhibited by WWL70 [23]–[24], THL [18], and also by JZL195 at the used concentration; BAT5, inhibited by THL [4], [18]. Note that palmostatin B dose-dependently inhibits probe labeling of BAT5, ABHD12 and LYPLA1/2, two closely-related proteins migrating at ∼25 kDa [25]. At the highest concentration, probe labeling of MAGL and ABHD6 is also inhibited. Note the absence of additional visible targets for palmostatin B at the tested concentrations. Data are representative from three ABPP experiments with similar outcome.

Palmostatin B was originally developed as an inhibitor of lysophospholipase 1 (LYPLA1), also known as acyl-protein thioesterase 1 (APT1) [19]–[20]. In the original report, selectivity over phospholipases such as PLA_1_, PLA_2_, PLD, and PLCβ was demonstrated [19]. As far as we are aware, however, the selectivity of palmostatin B has not been comprehensively evaluated. To explore the selectivity among the metabolic serine hydrolases, we conducted competitive ABPP with mouse brain membrane proteome. These studies revealed that palmostatin B (tested concentration range 10^−8^–10^−5^ M) dose-dependently inhibited probe binding to BAT5, ABHD12 and LYPLA1/2 (Figure 8). At the highest concentration, palmostatin B additionally inhibited probe binding to ABHD6 and MAGL. To complement the ABPP approach, hydrolase assays were performed using lysates or membrane preparations of human endocannabinoid hydrolase expressing cells with the outcome that palmostatin B potently inhibited hABHD6 (IC_50_ ∼50 nM) and hMAGL (IC_50_ ∼90 nM). At higher concentrations it also inhibited hABHD12 (IC_50_ ∼2 µM) whereas fatty acid amide hydrolase (hFAAH) was resistant to this inhibitor (Table 1). In addition, ebelactone A and hymeglusin inhibited hABHD6 with moderate potencies (IC_50_ ∼0.9 and 3.2 µM, respectively) whereas hABHD12, hMAGL and hFAAH were resistant to these β-lactones.

**Table 1:** Sensitivity of hABHD6, hMAGL, hABHD12 and hFAAH towards palmostatin B and related β-lactones.

	Palmostatin B	THL	Ebelactone A	Hymeglusin
hABHD6-HEKlysates	–7.28±0.05(52.5 nM)	–7.32±0.06#(47.9 nM)	–6.06±0.02(0.87 µM)	–5.50±0.04(3.2 µM)
hMAGL-HEKlysates	–7.03±0.06(93.3 nM)	Remaining activity,88.1±6.6% at 10^−5^ M	Remaining activity,90.8±6.7%at 10^−5^ M	Remaining activity,105.7±0.3%at 10^−5^ M
hABHD12-HEKlysates	–5.74±0.06(1.8 µM)	–6.72±0.07#(192.8 nM)	Remaining activity,100.5±6.4%at 10^−5^ M	Remaining activity,102.8±0.5%at 10^−5^ M
hFAAH-COS7membranes	Remainingactivity, 92.4±1.3% at10^−6^ M	Remaining activity,100.3±1.3%at 10^−5^ M	Remaining activity,87.3±3.1%at 10^−5^ M	Remaining activity,79.2±2.6% at10^−5^ M

# Data are from reference [10].

Hydrolase activities were determined using lysates of HEK293 cells with transient expression of hABHD12, hABHD6 and hMAGL or membranes of hFAAH expressing COS-7 cells, as previously described [10], [30]. Inhibitor dose–response curves were determined and the IC_50_ values calculated as non-linear regressions using GraphPad Prism 5.0 for Windows. Data are presented as log[IC_50_] values (mean ± SEM) from 3 independent experiments. The IC_50_ values are shown in parenthesis.

Collectively these studies demonstrate that palmostatin B hits several targets among the metabolic serine hydrolase family, raising potential concerns regarding the utility of this compound as a specific LYPLA1 inhibitor. It is noteworthy that the potencies of palmostatin B to inhibit hABHD6 and hBAT5 were ∼13- and ∼7-fold higher, respectively than the IC_50_ value (670 nM) originally reported for this compound *in vitro* towards LYPLA1 [19]. The potency obtained here for THL is roughly comparable with that (IC_50_ 30 nM) previously reported for THL towards recombinant hBAT5 in competitive ABPP experiments [4]. Under the presently used conditions, THL was previously reported to inhibit hABHD6 and hABHD12 with IC_50_ values of ∼50 and ∼190 nM, respectively [10].

### 1,3,4-Oxadiazol-2(3H)-ones as BAT5 inhibitors

**Figure 9:**
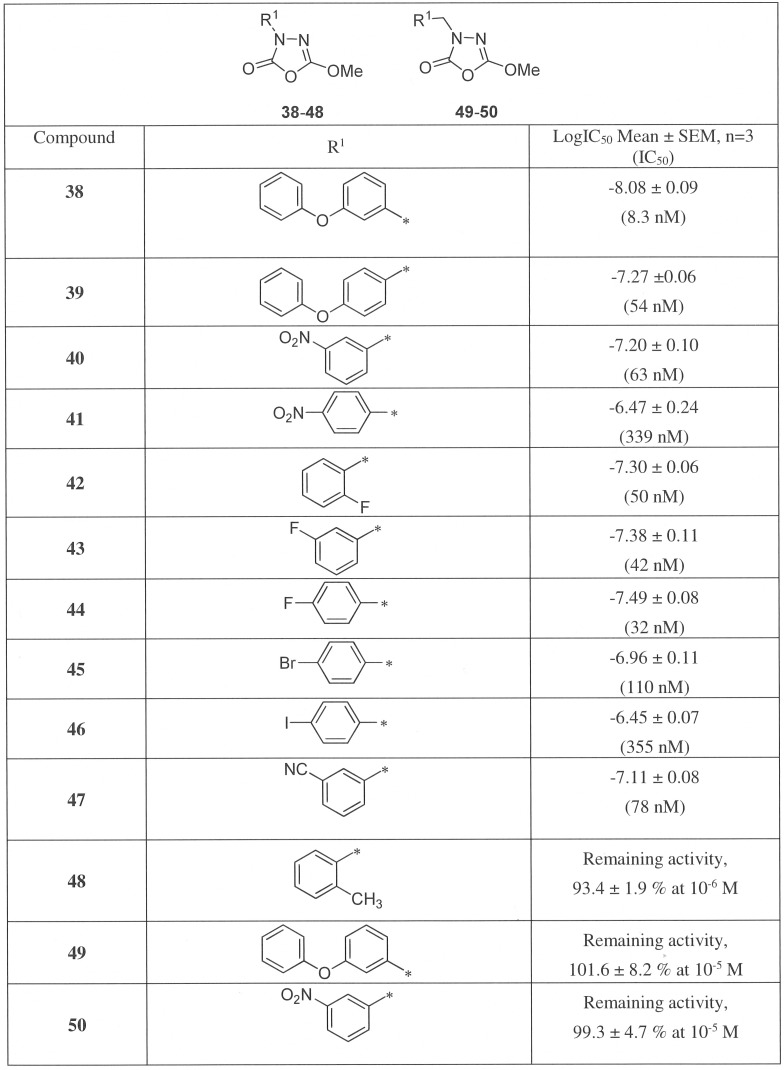
Inhibition of hBAT5 by phenyl and benzyl substituted 1,3,4-oxadiazol-2(3H)-ones 38–50. Lysates of hBAT5-HEK293 cells were treated for 30 min with the inhibitors after which glycerol liberated from 1-LG hydrolysis (25 µM final concentration) was kinetically monitored for 90 min. Inhibitor dose–response curves and IC_50_ values derived thereof were calculated from non-linear regressions using GraphPad Prism 5.0 for Windows. Values are mean ± SEM from three independent experiments using lysates from one bacth of transfection.

As our competitive ABPP experiments indicated that the β-lactones showed poor selectivity among the serine hydrolases, we explored additional chemical scaffolds commonly present in general lipase inhibitors as potential inhibitors of BAT5 activity. We noted that the hormone-sensitive lipase (HSL) inhibitor C7600 (**38** in our compound series) was a potent inhibitor of hBAT5-mediated 1-LG hydrolysis (IC_50_ 8.3 nM) (Figure 9). It is noteworthy that the reported *in vitro* potency for HSL inhibition was 70 nM [26], being almost 10-fold lower than the value observed here for hBAT5. However, **38** should be considered as a broadly acting lipase inhibitor [27] and our previous studies reported that the compound inhibited FAAH (IC_50_ ∼6 nM) and MAGL (IC_50_ ∼0.4 µM) [28]–[29]. Therefore in efforts to increase selectivity towards BAT5, we made structural modifications to the 1,3,4-oxadiazol-2(3H)-one backbone of **38** and collected the SAR data for such compounds as hBAT5 inhibitors.

We tested several phenyl and benzyl substituted 1,3,4-oxadiazol-2(3H)-ones by varying the substituents of the 5-phenyl ring (Figure 9). The general observation was that electron withdrawing substituents in the phenyl ring led to enhanced hBAT5 inhibition potencies. The 3-phenoxyphenyl analogue **38** was over 6-fold more potent than its 4-phenoxyphenyl counterpart **39**. The same preference for *meta*-substitution was seen for the nitro-substituted analogues **40** (IC_50_ 63 nM) and **41** (IC_50_ 339 nM). The compounds containing electron withdrawing fluorine in *ortho*-, *meta*- and *para*-positions (**42–44**) of the phenyl ring showed nanomolar hBAT5 inhibition and were all almost equipotent (IC_50_ 50–32 nM).

Among the *para*-halogen substituted analogues, the potency rank order of IC_50_ values was: **44** (p-F, 32 nM) <**45** (p-Br, 110 nM) <**46** (p-I, 355 nM), that is, the inhibitory activity was decreased with increasing size of halogen. As expected, the 3-cyano substituted analogue **47** exhibited good hBAT5 inhibition (IC_50_ 78 nM), demonstrating again that the presence of electron withdrawing substituent at the *meta*-position was beneficial for the inhibition. Interestingly, the *ortho*-methyl analogue **48** was inactive suggesting that electron-releasing methyl group at the *ortho*-position diminished the binding affinity to the hBAT5 enzyme. Replacement of the 3-phenyl with a benzyl (analogues **49** and **50**) resulted in loss of hBAT5 inhibitory activity, indicating that the introduction of a methylene spacer between the aromatic ring and oxadiazolone core was not tolerated.

**Figure 10:**
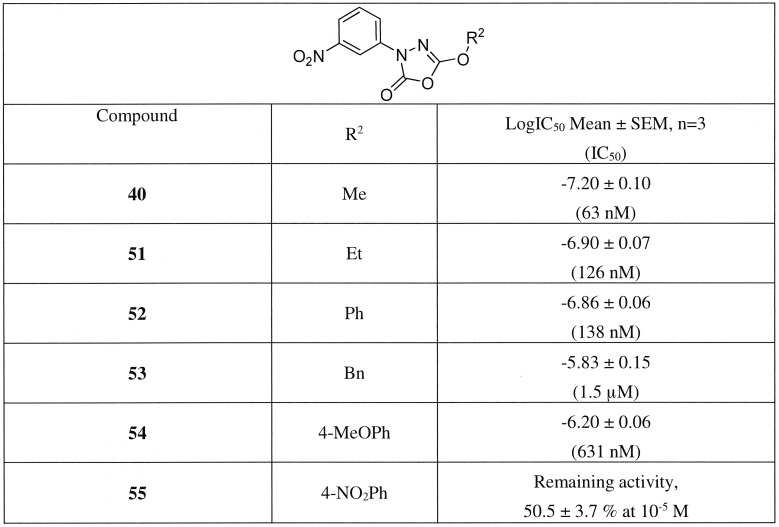
Inhibition of hBAT5 by 5-alkoxy and phenoxy substituted 3-(3-nitrophenyl)-1,3,4-oxadiazol-2(3H)-ones 40, 51–55. Lysates of hBAT5-HEK293 cells were treated for 30 min with the inhibitors after which glycerol liberated from 1-LG hydrolysis (25 µM final concentration) was kinetically monitored for 90 min. Inhibitor dose–response curves and IC_50_ values derived thereof were calculated from non-linear regressions using GraphPad Prism 5.0 for Windows. Values are mean ± SEM from three independent experiments using lysates from one bacth of transfection.

Finally, we investigated the effects of different alkoxy and phenoxy substituents at the 5-position of the oxadiazolone core (Figure 10), starting with the replacement of the methoxy group with ethyl, phenyl, benzyl and 4-substituted phenyl groups (**51–55**). We observed that the inhibitor potency was decreased with increasing size of the substituent. The activity was decreased about twofold when methyl was replaced with ethyl (**51**) or phenyl (**52**). The benzyl substitution (**53**) decreased activity 24-fold, the 4-methoxyphenyl (**54**) 10-fold and the 4-nitrophenyl substituent analogue (**55**) was the weakest inhibitor of this series.

Collectively, this preliminary SAR analysis indicated that the small electron withdrawing substituent or larger substituents (OPh, NO_2_, CN) at the *meta*-position were favorable for hBAT5 inhibition. Additionally, we have shown that a small substituent (methoxy) at the 5-position and direct attachment of an aromatic ring at the 3-position of the 1,3,4-oxadiazol-2-one ring were optimal for the hBAT5 inhibitory activity.

**Figure 11:**
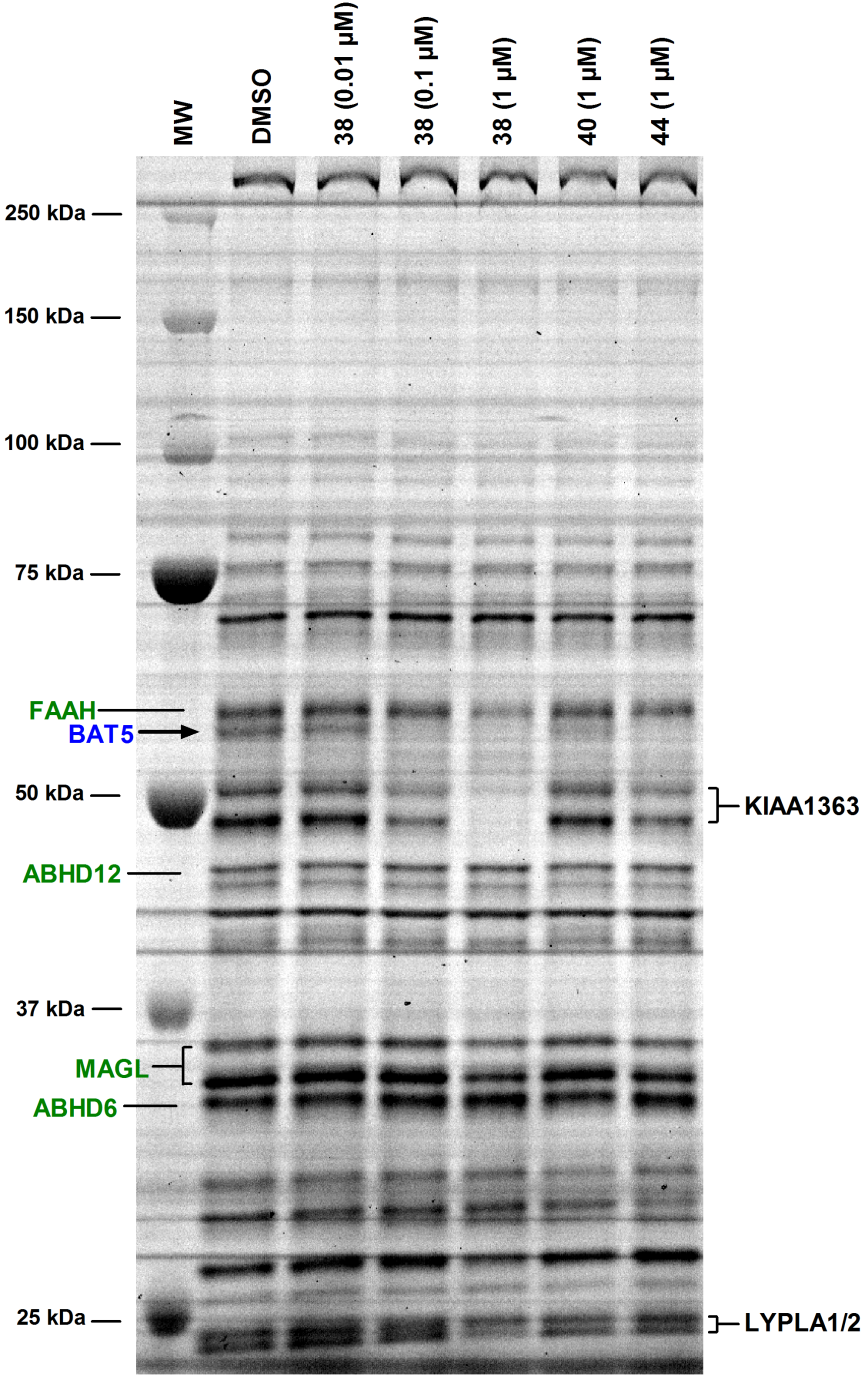
Competetive ABPP profiling of the selectivity of the HSL inhibitor C7600 (38) and its analogues 40 and 44 among the serine hydrolases in mouse wholebrain membrane proteome. Membranes (100 µg) were treated either with DMSO or the indicated concentrations of the inhibitors for 60 min at RT. After this, serine hydrolases were labeled for 60 min using the active site serine targeting fluorescent probe TAMRA-FP. After separation in SDS-electrophoresis gel (10%), serine hydrolases were visualized by in-gel fluorescent gel scanning as detailed in the Methods section. Molecular weight markers (MW) are indicated at left. Consistent with previous reports [28]–[29], C7600 (**38**) targets the endocannabinoid hydrolases FAAH and MAGL, as evidenced by dose-dependent inhibition of probe binding to these serine hydrolases. In addition, **38** inhibits probe labeling of KIAA1363, a protein doublet migrating at ∼50 kDa [4] and of LYPLA1/2, two closely-related proteins migrating at ∼25 kDa [25]. Note sensitivity of BAT5 to all tested inhibitors. Note also improved BAT5 selectivity of **40** and **44** as compared to **38**. The gel is representative from two (four in the case of **40**) independent ABPP experiments with similar outcome.

As the final step in these studies, competitive ABPP was used to evaluate the BAT5 selectivity of compounds **38** (C7600), **40** and **44** among the serine hydrolases of mouse brain membrane proteome. These experiments indicated that at 1 µM concentration, **38** targeted FAAH, BAT5, KIAA1363, MAGL and LYPLA1/2 (Figure 11). In contrast, the *meta*-nitro-substituted analogue **40** showed improved BAT5 selectivity as it did not visibly affect probe binding to FAAH, KIAA1363, MAGL or LYPLA1/2, the off-targets of **38** (Figure 11). Similarly, **44** showed improved BAT5 selectivity as compared to **38**, although at 1 µM concentration this compound shared one off-target with **38**, namely KIAA1363.

## Conclusions

In an attempt to provide the initial biochemical and pharmacological characterization of BAT5 *in vitro*, this study has revealed for the first time the substrate and inhibitor preferences of this unannotated ABHD family member of metabolic serine hydrolases. By combining a powerful activity-based chemoproteomic approach with sensitive hydrolase assays that previously facilitated characterization of related ABHD family members, we verified successful production of catalytically active BAT5 in HEK293 cells, enabling more detailed biochemical characterization. The current work provides evidence that BAT5 acts as a genuine MAG lipase with preference for medium-chain saturated (C14∶0) and long-chain unsaturated (C18∶1, C18∶2, C20∶4) MAGs as well as for one particular PG-G. Only marginal activity was detected towards the tested DAG, TAG and lysophospholipid species. A lipase-like role was further supported by the identification of known lipase inhibitors, such as C7600, palmostatin B, and THL, as potent inhibitors of BAT5 activity. Besides BAT5, palmostatin B was demonstrated to potently target additional members of the serine hydrolase family, a finding raising concerns regarding the utility of this compound as a specific LYPLA1 inhibitor. Structural modification of C7600 yielded compounds with improved BAT5 selectivity. Collectively these findings suggest that BAT5 could function as a MAG lipase *in vivo* as well and in this capacity may regulate glycerolipid metabolism in tissues enriched with this enzyme. However, several important questions remain, one obvious being whether and how adequately the presently described substrate profile reflects the situation *in vivo?* Also, why are there multiple functional motifs conserved in the BAT5 primary structure? What specific roles do the two fully conserved lipase-like motifs play? Does BAT5 mediate also acyltransferase activity, as suggested by the presence of the HxxxxD motif? Future studies should aim at addressing these questions.

## Methods

### Drugs, chemicals and reagents

All reagents for the fluorescent glycerol assay, THL (orlistat), hymeglusin, ebelactone A, and the following substrates were purchased from Sigma (St. Louis, MO): 1-oleyl-2-hydroxy-*sn*-glycero-3-phosphate (C18∶1-LPA), 1-capryloyl-*rac*-glycerol (1-CG) [C8∶0], 1-decanoyl-*rac*-glycerol (1-DG) [C10∶0], 1-lauroyl-*rac*–glycerol (1-LaurG) [C12∶0], 1-myristoyl-*rac*–glycerol (1-MG) [C14∶0], 2-palmitoyl-*rac*–glycerol (2-PG) [C16∶0], and the 1- and 2-oleoylglycerol (1- and 2-OG) [C18∶1]. The inhibitors IDFP and MAFP, the 1- and 2-linoleoylglycerol (1- and 2-LG) [C18∶2] and the 1- and 2-arachidonoylglycerol (1- and 2-AG) [C20∶4], as well as prostaglandin D_2_-1-glycerol ester (PGD_2_-G), prostaglandin E_2_-1-glycerol ester (PGE_2_-G), prostaglandin F_2α_-1-glycerol ester (PGF_2α_-G) and 15-deoxy-Δ^12,14^-prostaglandin J_2_-2-glycerol ester (15d-PGJ_2_-G) were from Cayman Chemicals (Ann Arbor, MI). In inhibitor profiling experiments, 1-LG from Sigma was used as the substrate as it was most cost-effective. Lysophosphatidylserine (C18∶1-LPS) was purchased from Avanti Polar Lipids (Alabaster, AL, USA) and palmostatin B and HDSF from Calbiochem (EMD Millipore). TAMRA-FP was obtained from Thermo Fisher Scientific (Rockford, IL). All the other reagents were of highest purity available.

### Chemistry

**Figure 12:**
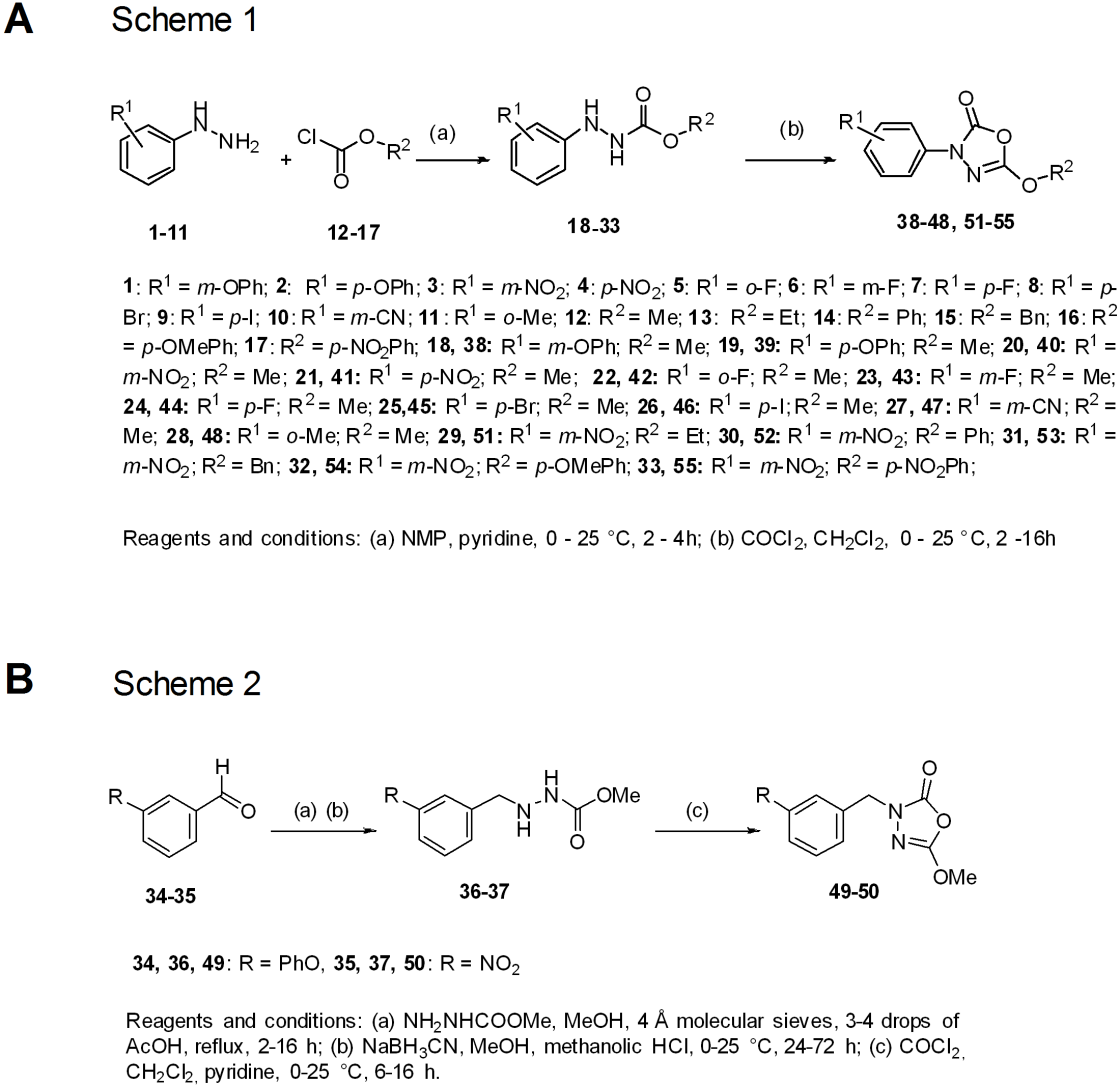
Schemes outlining the synthesis procedures for the C7600 analogues of this study.

The 3-phenyl-1,3,4-oxadiazol-2(*3H*)-ones **38–48**, **51–55** were prepared according to Minkkilä et al. [29] from appropriate hydrazines (**1–11**) and chloroformates (**12–17**) to afford hydrazine carboxylates (**18–33**) followed by cyclization with phosgene in presence of base as outlined in Scheme 1 (Figure 12A).

The 3-benzyl-1,3,4-oxadiazol-2-ones (Scheme 2, Figure 12B) were prepared according to Patel et al. [24] by condensation of an appropriate aldehyde (**34–35**) with methyl carbazate to afford Schiff’s base which was reduced to the corresponding hydrazine carboxylate derivative (**36–37**) and subsequently cyclized with phosgene to 1,3,4-oxadiazolone (**49–50)**.

### General chemical procedures

^1^H and ^13^C NMR spectra were recorded on a Bruker Avance instrument operating at 500.1 and 25.8 MHz, respectively. Chemical shifts are reported as d values (ppm) relative to an internal standard of tetramethylsilane (TMS). The following abbreviations were used to explain multiplicities: br, broad; d, doublet; m, multiplet; q, quartet; s, singlet; t, triplet. Electrospray ionization mass spectra were determined on Finnigan MAT LCQ quadrupole ion trap mass spectrometer. Elemental analyses were performed on a ThermoQuest CE instrument (EA 1110 CHNS-O) or a Perkin-Elmer PE 2400 Series II CHNS-O Analyser. All chemicals and solvents were of commercial quality and were used without further purification. Most intermediate and end products were purified by flash chromatography using 30–60 µm silica gel and an appropriate eluent.

### Synthesis procedures for the preparation of hydrazine carboxylates 20, 21, 29–33 and 37 and 1,3,4-oxadiazol-2-ones 40, 41, 50 and 51–55

Methyl hydrazine carboxylates (**18**, **19**, **22–28**) and 1,3,4-oxadiazol-2-ones (**38**, **39**, **42–49**) were synthesized as described previously [24], [28].

### Methyl 2-(3-nitrophenyl)hydrazinecarboxylate (20)

Orange solid (450 mg, 82%); ^1^H NMR (CDCl_3_): *δ* 7.61–7.80 (m, 2H), 7.38 (t, *J* = 8.04 Hz, 1H), 7.07–7.18 (m, 1H), 6.71 (br s, 1H), 6.09 (br s, 1H), 3.79 (s, 3H).

### Methyl 2-(4-nitrophenyl)hydrazinecarboxylate (21)

Orange solid (1.5 g, 72%); ^1^H NMR (CDCl_3_): *δ* 8.18 (d, *J* = 8.83 Hz, 2H), 6.88 (d, *J* = 9.14 Hz, 2H), 6.61 (br s, 1H), 6.23 (br s, 1H), 3.82 (s, 3H).

### Ethyl 2-(3-nitrophenyl)hydrazinecarboxylate (29)

Yellow solid (662 mg, 90%); ^1^H NMR (CDCl_3_): *δ* 7.71 (d, *J* = 8.20 Hz, 1H), 7.66 (s, 1H), 7.35 (t, *J* = 8.04 Hz, 1H), 7.02–7.17 (m, 1H), 6.65 (br s, 1H), 6.18 (br s, 1H), 4.22 (q, *J* = 6.94 Hz, 2H), 1.14–1.33 (m, 3H).

### Phenyl 2-(3-nitrophenyl)hydrazinecarboxylate (30)

Orange solid (790 mg, 89%); ^1^H NMR (CDCl_3_): *δ* 7.65–7.78 (m, 2H), 7.37 (d, *J* = 4.10 Hz, 3H), 7.23 (s, 1H), 7.08–7.20 (m, 3H), 7.03 (br s, 1H), 6.25 (br s, 1H).

### Benzyl 2-(3-nitrophenyl)hydrazinecarboxylate (31)

Yellow solid (1.3 g, 46%); ^1^H NMR (CDCl_3_): *δ* 7.57–7.79 (m, 2H), 7.29–7.46 (m, 6H), 7.07 (d, *J* = 7.88 Hz, 1H), 6.76 (br s, 1H), 6.19 (br s, 1H), 5.18 (s, 2H).

### 4-Methoxyphenyl 2-(3-nitrophenyl)hydrazinecarboxylate (32)

Yellow solid (1.9 g, 96%); ^1^H NMR (CDCl_3_): *δ* 7.67–7.79 (m, 2H), 7.37 (t, *J* = 8.04 Hz, 1H), 7.14 (dd, *J* = 8.20, 1.58 Hz, 1H), 7.07 (br s, 1H), 6.94–7.05 (m, 2H), 6.87 (d, *J* = 8.51 Hz, 2H), 6.24 (br s, 1H), 3.78 (s, 3H).

### 4-Nitrophenyl 2-(3-nitrophenyl)hydrazinecarboxylate (33)

Yellow solid (675 mg, 65%); ^1^H NMR (CDCl_3_): *δ* 10.18 (s, 1H), 8.62 (s, 1H), 8.30 (d, *J* = 8.83 Hz, 2H), 7.54–7.65 (m, 2H), 7.43–7.54 (m, 3H), 7.23 (d, *J* = 7.88 Hz, 1H).

### Methyl 2-(3-nitrobenzyl)hydrazinecarboxylate (37)

Colourless oil (3.17 g, 71%); ^1^H NMR (CDCl_3_): *δ* 8.24 (s, 1H), 8.14 (d, *J* = 8.5 Hz, 1H), 7.68 (d, *J* = 7.5 Hz, 1H), 7.53–7.49 (dd, *J* = 13.1 & 5.0 Hz, 1H), 6.21 (br s, 1H), 4.38 (br s, 1H), 4.13 (s, 2H), 3.76 (s, 3H).

### 5-Methoxy-3-(3-nitrophenyl)-1,3,4-oxadiazol-2(3H)-one (40)

White solid (110 mg, 22%); ^1^H NMR (CDCl_3_): *δ* 8.62 (d, *J* = 2.21 Hz, 1H), 8.20–8.31 (m, 1H), 8.03–8.12 (m, 1H), 7.61 (t, *J* = 8.20 Hz, 1H), 4.17 (s, 3H); ^13^C NMR (CDCl_3_): *δ* 156.22, 148.78, 147.95, 137.18, 130.20, 122.97, 119.96, 112.81, 58.11; ESI-MS: 238.09 [M + H]^+^.

### 5-Methoxy-3-(4-nitrophenyl)-1,3,4-oxadiazol-2(3H)-one (41)

White solid (401 mg, 71%); ^1^H NMR (CDCl_3_): *δ* 8.34 (d, *J* = 9.46 Hz, 2H) 8.04 (d, *J* = 9.14 Hz, 2H) 4.19 (s, 3H); ^13^C NMR (CDCl_3_): *δ* 156.09, 147.79, 144.24, 140.86, 124.73 (2C), 117.32 (2C), 57.84; ESI-MS: 238.09 [M + NH_4_]^+^.

### 5-Methoxy-3-(3-nitrobenzyl)-1,3,4-oxadiazol-2(3H)-one (50)

Colourless oil (965 mg, 28%); ^1^H NMR (CDCl_3_, 500 MHz): *δ* 8.22 (s, 1H), 8.21 (d, *J* = 9.0 Hz, 1H), 7.69 (d, *J* = 7.5 Hz, 1H), 7.57 (t, *J* = 7.75 Hz, 1H), 4.88 (s, 2H), 3.97 (s, 3H); ^13^C NMR (CDCl_3_): *δ* 155.85, 151.29, 148.49, 136.89, 134.20, 129.91, 123.36, 123.17, 57.53, 48.59; Anal. Calcd for C_10_H_9_N_3_O_5_: C, 47.81; H, 3.61; N, 16.73%. Found: C, 47.77; H, 3.58; N, 15.98%; ESI-MS: 252.03 [M + H]^+^.

### 5-Ethoxy-3-(3-nitrophenyl)-1,3,4-oxadiazol-2(3H)-one (51)

White solid (448 mg, 62%); ^1^H NMR (CDCl_3_): *δ* 8.60 (s, 1H), 8.19–8.31 (m, 1H), 8.00–8.12 (m, 1H), 7.60 (t, *J* = 8.20 Hz, 1H), 4.53 (q, *J* = 6.94 Hz, 2H), 1.53 (t, *J* = 7.09 Hz, 3H); ^13^C NMR (CDCl_3_): *δ* 155.51, 148.77, 147.98, 137.23, 130.23, 122.96, 119.86, 112.77, 68.32, 14.32; Anal. Calcd for C_10_H_9_N_3_O_5_: C, 47.81; H, 3.61; N, 16.73%. Found: C, 47.85; H, 3.62; N, 16.52%; ESI-MS: 252.25 [M + H]^+^.

### 3-(3-Nitrophenyl)-5-phenoxy-1,3,4-oxadiazol-2(3H)-one (52)

White solid (312 mg, 36%); ^1^H NMR (CDCl_3_): *δ* 8.57 (s, 1H), 8.13–8.22 (m, 1H), 8.01–8.11 (m, 1H), 7.59 (t, *J* = 8.35 Hz, 1H), 7.44–7.54 (m, 2H), 7.30–7.44 (m, 3H); ^13^C NMR (CDCl_3_): *δ* 154.54, 151.44, 148.77, 147.67, 136.94, 130.27, 130.17, 127.18, 123.22 (2C), 120.25 (2C), 119.62, 112.98; Anal. Calcd for C_14_H_9_N_3_O_5_: C, 56.19; H, 3.03; N, 14.04%. Found: C, 56.09; H, 3.03; N, 13.82%; ESI-MS: 300.37 [M + H]^+^.

### 5-(Benzyloxy)-3-(3-nitrophenyl)-1,3,4-oxadiazol-2(3H)-one (53)

White solid (940 mg, 66%); ^1^H NMR (CDCl_3_): *δ* 8.62 (s, 1H), 8.19–8.30 (m, 1H), 8.07 (d, *J* = 7.88 Hz, 1H), 7.61 (t, *J* = 8.20 Hz, 1H), 7.35–7.55 (m, 5H), 5.45 (s, 2H); ^13^C NMR (CDCl_3_): *δ* 155.44, 148.80, 147.92, 137.19, 132.92, 130.26, 129.63, 128.94, 122.99, 119.95, 112.84, 110.01, 73.47; Anal. Calcd for C_15_H_11_N_3_O_5_: C, 57.51; H, 3.54; N, 13.41%. Found: C, 57.50; H, 3.54; N, 13.31%; ESI-MS: 314.39 [M + H]^+^.

### 5-(4-Methoxyphenoxy)-3-(3-nitrophenyl)-1,3,4-oxadiazol-2(3H)-one (54)

White solid (312 mg, 15%); ^1^H NMR (CDCl_3_): *δ* 8.55 (t, *J* = 2.05 Hz, 1H), 8.17 (dd, *J* = 8.35, 1.42 Hz, 1H), 8.06 (dd, *J* = 8.20, 1.58 Hz, 1H), 7.58 (t, *J* = 8.20 Hz, 1H), 7.19–7.40 (m, 2H), 6.88–7.08 (m, 2H) 3.85 (s, 3H); ^13^C NMR (CDCl_3_): *δ* 158.20, 155.04, 148.75, 147.73, 144.87, 136.97, 130.17, 123.21, 120.78 (2C), 120.16, 115.01 (2C), 112.94, 55.74; Anal. Calcd for C_15_H_11_N_3_O_6_: C, 54.72; H, 3.37; N, 12.76%. Found: C, 55.07; H, 3.38; N, 12.79%; ESI-MS: 330.39 [M + H]^+^.

### 5-(4-Nitrophenoxy)-3-(3-nitrophenyl)-1,3,4-oxadiazol-2(3H)-one (55)

White solid (312 mg, 43%); ^1^H NMR (CDCl_3_): *δ* 8.59 (s, 1H), 8.43 (d, *J* = 9.14 Hz, 2H), 8.16–8.27 (m, 1H), 8.08–8.16 (m, 1H), 7.59–7.73 (m, 3H); ^13^C NMR (CDCl_3_): *δ* 155.27, 153.47, 148.81, 147.16, 146.11, 136.62, 130.41, 126.09 (2C), 123.22, 120.64, 120.33, 112.99 (2C); Anal. Calcd for C_14_H_8_N_4_O_7_: C, 48.85; H, 2.34; N, 16.28%. Found: C, 48.81; H, 2.32; N, 15.92%; ESI-MS: 345.28 [M + H].

### Generation of HEK293 cells with transient expression of BAT5

The procedures were as previously described for generation of the endocannabinoid hydrolase expressing cell lines [10]. Briefly, HEK293-cells were cultured as monolayers in DMEM (Euroclone) containing 10% fetal bovine serum (Euroclone), under antibiotics (penicillin/streptomycin, Euroclone) at 37°C in a humidified atmosphere of 5% CO_2_/95% air. Fully sequenced plasmids containing hBAT5 or mBAT5, obtained from Origene Technologies Inc. (Rockville, MD), were introduced to cells by a standard (transient) transfection procedure using X-tremeGENE Hp DNA Transfection reagent (Roche, Mannheim, Germany) following manufacturer’s instructions. Cellular lysates were prepared and protein concentrations were measured as previously described [10].

### Hydrolase activity assays

Glycerol liberated from the hydrolysis of glycerolipid substrates was determined kinetically using a fluorometric assay, essentially as previously described [10]. Briefly, glycerol production was coupled via a three-step enzymatic cascade to hydrogen peroxide (H_2_O_2_) dependent generation of resorufin whose fluorescence (λ_ex_ 530; λ_em_ 590 nm) was kinetically monitored using a Tecan Infinite M200 plate reader (Tecan Group Ltd., Männedorf, Switzerland). The assays routinely contained 0.5% (w/v) BSA (essentially fatty acid free) as a carrier for lipophilic compounds. The BAT5 substrate preference was determined by monitoring glycerol production in lysates of hBAT5-HEK293 cells in assay mixes containing MAG, DAG or TAG substrates (25 µM final concentration) with varying acyl chain length and saturation, as detailed in Figure 3. The twelve MAG substrates were tested also in assays without BSA. In addition, four commercially available PG-Gs were included. To monitor assay performance, assay blanks without enzyme, cellular background (HEK293/Mock cell lysates) as well as a glycerol standard were included for each tested substrate. Fluorescence of the assay blank was subtracted before calculation of the final results. Inhibitory activity towards FAAH was determined using membranes of hFAAH overexpressing COS-7 cells, as previously described [30]. The final incubation volume (100 µl) contained 1 µg of protein and the substrate concentration was 20 µM (10 nM of ^3^H-anandamide having specific activity of 60 Ci/mmol and concentration of 1 mCi/ml).

### Determination of fatty acid release

We adapted the Free Fatty Acid Assay Kit (Cayman Chemical Item Number 700310) to kinetically monitor fatty acid release from selected substrates using lysates of HEK293 cells (0.3 µg/well) transiently expressing hBAT5 or hABHD12 (as a positive control). The assay protocol was tailored to closely follow the scheme of the 96-well-plate glycerol assay with the exception that BSA was omitted. Briefly, fatty acid production was coupled via a three-step enzymatic cascade to hydrogen peroxide (H_2_O_2_) dependent generation of resorufin whose fluorescence was monitored as described above for the glycerol assay. The substrates were tested at 25 µM final concentration and for validation purposes included the MAGs 1-AG and 1-LG, the lysophospholipids C18∶1-LPA and C18∶1-LPS, the DAG 1,2-dioleoyl(C18∶1)-*rac-*glycerol, and the TAG 1,2,3-trioleoyl(C18∶1)glycerol. For each tested substrate, assay blank without enzyme and a fatty acid standard (C18∶1-FA, 500 pmol/well) was included. Fluorescence of the assay blank was subtracted before calculation of the final results.

### Activity-based protein profiling (ABPP) of serine hydrolases

Competitive ABPP using mouse whole brain membranes or HEK293 cell lysates was conducted to visualize the effect of selected inhibitors on BAT5 activity in native brain membrane proteome and in lysates of HEK293 cells with transient expression of hBAT5 or mBAT5. We used the active site serine-targeting fluorescent fluorophosphonate probe TAMRA-FP as previously described [10]. Briefly, lysates (25 µg) of HEK293 cells with or without BAT5 overexpression or whole mouse brain membranes (100 µg) were treated for 1 h with DMSO or the selected inhibitors, after which TAMRA-FP labelling was conducted for 1 hour at RT (final probe concentration 2 µM). The reaction was quenched by addition of 2xgel loading buffer, after which 10 µg protein (10 µl) was loaded per lane and the proteins were resolved in 10% SDS-PAGE together with molecular weight standards. TAMRA-FP labeling was visualized (λ_ex_ 552; λ_em_ 575 nm) by a fluorescent reader (FLA-3000 laser fluorescence scanner, Fujifilm, Tokyo, Japan). The intensity of bands was quantified using ImageJ, a freely available image analysis software (http://rsbweb.nih.gov/ij/).

### Data reproducibility and statistical analyses

For hBAT5 and mBAT5, transient transfections were repeated twice and the major findings confirmed using lysates from both batches. A more comprehensive characterization was performed using one particular batch of the human enzyme. With the exception of the FFA data presented in Figure 4, all numerical data are mean ± SEM from at least three independent experiments. The K_m_ and V_max_ values, inhibitor dose–response curves and IC_50_ values derived thereof were calculated from non-linear regressions using GraphPad Prism 5.0 for Windows. Statistical comparisons were done by using either paired or unpaired t-test, depending on study design as detailed in the legends of individual figures. The significance is denoted with an asterix (*, p<0.05; **, p<0.01 and ***p<0.001).

### Ethics Statement

For the ABPP experiments *in vitro* with native mouse brain membrane proteome, membranes prepared from brain tissue of 4-week-old male mice were used. The animals were obtained from the National Laboratory Animal Centre, University of Eastern Finland. The mice were bred, housed, and sacrificed for the sole purposes of the experiment. The animals were sacrificed using decapitation. Approval for the harvesting of animal tissue was applied, registered and obtained from the local welfare officer of the University of Eastern Finland. No further ethical approval was required, as the experiments did not involve any *in vivo* treatment.

## Supporting Information

Figure S1 **Background activity in HEK293 (A) and Mock-transfected cells (B) towards the 19 substrates tested in this study.** The substrate panel included monoacylglycerols (MAGs) with the indicated acyl chain length, isomer and degree of saturation, the diacylglycerol (DAG) 1,2-dioleoyl(C18∶1)-*rac-*glycerol, the triacylglycerols (TAG-1 = 1,2,3-trioleoyl(C18∶1)glycerol; TAG-2 = 1-palmitoyl(C16∶0)-2-oleoyl(C18∶1)-3-linoleoyl(C18∶2)-*rac*-glycerol, as well as the prostaglandin glycerol esters PGD_2_-G, PGE_2_-G, PGF_2α_-G and 15d-PGJ_2_-G. Cellular lysates (0.3 µg/well) were incubated together with the indicated substrates [25 µM final concentration, added from 10 mM stock solutions in ethanol into the glycerol assay mix containing 0.5% (w/v) fatty acid free BSA and 1% (v/v ethanol). Glycerol production was determined at time-point 60 min. Statistical comparisons (unpaired t-test) indicated no significant differences (p>0.05) in the activity between HEK and Mock cells towards any tested substrate.
(TIF)

Figure S2 **hBAT5-catalyzed hydrolysis of LPS or TAG does not exceed cellular background activity.** Shown is time-dependency of fluorescence resulting from fatty acid liberation in incubations of the indicated substrates together with lysates of HEK293 cells with or without overexpression of hBAT5 or hABHD12. HEK293 cells were transiently transfected with the cDNA encoding hBAT5 or hABHD12, as detailed in [10] and the Methods section. After 48 h, cells were harvested and lysates prepared for lipase activity measurements based on the Cayman’s FFA fluorescence assay kit. The substrates included the MAGs 1-AG (**A**) and 1-LG (**B**), the lysophospholipid C18∶1-LPS (**C**) and the TAG 1,2,3-trioleoyl(C18∶1)glycerol (**D**). Cellular lysates (0.3 µg/well) were incubated together with the indicated substrates [50 µM final concentration, added from 10 mM stock solutions in ethanol into the FFA assay mix containing 0.1% (v/v) ethanol. Each condition included also assay blanks lacking the lysate. Raw fluorescence readings are shown for time-points 0, 10 and 20 min. Data are mean ± SD from duplicate wells. Note absence of hBAT5-catalyzed hydrolysis of LPS and TAG, as evidenced by parallel lines representing Blank, HEK and hBAT5-HEK lysates. Note also that 1-AG and 1-LG are hydrolyzed by hBAT5 and hABHD12 at rates clearly exceeding the cellular background and that LPS is hydrolyzed by hABHD12.
(TIF)

Figure S3 **Lipophilicity vs. hBAT5 utilization of the twelve MAG species tested in this study using routine assay conditions with 0.5% (w/v) BSA included.** **A.** LogP values were calculated using Advanced Chemistry Development (ACD/Labs) Software V11.02 (© 1994–2014 ACD/Labs). **B.** Mean substrate utilization (data extracted from Fig. 3B) plotted against the LogP values. The slope of the best-fit line (dashed, obtained by linear regression analysis) does significantly deviate from zero.
(TIF)
