# Supplementary figures and images for: Biochemical and Pharmacological Characterization of the Human Lymphocyte Antigen B-Associated Transcript 5 (BAT5/ABHD16A)
Source: PLoS One. 2014 Oct 7;9(10):e109869. doi: 10.1371/journal.pone.0109869 (PMC4188605; doi:10.1371/journal.pone.0109869)

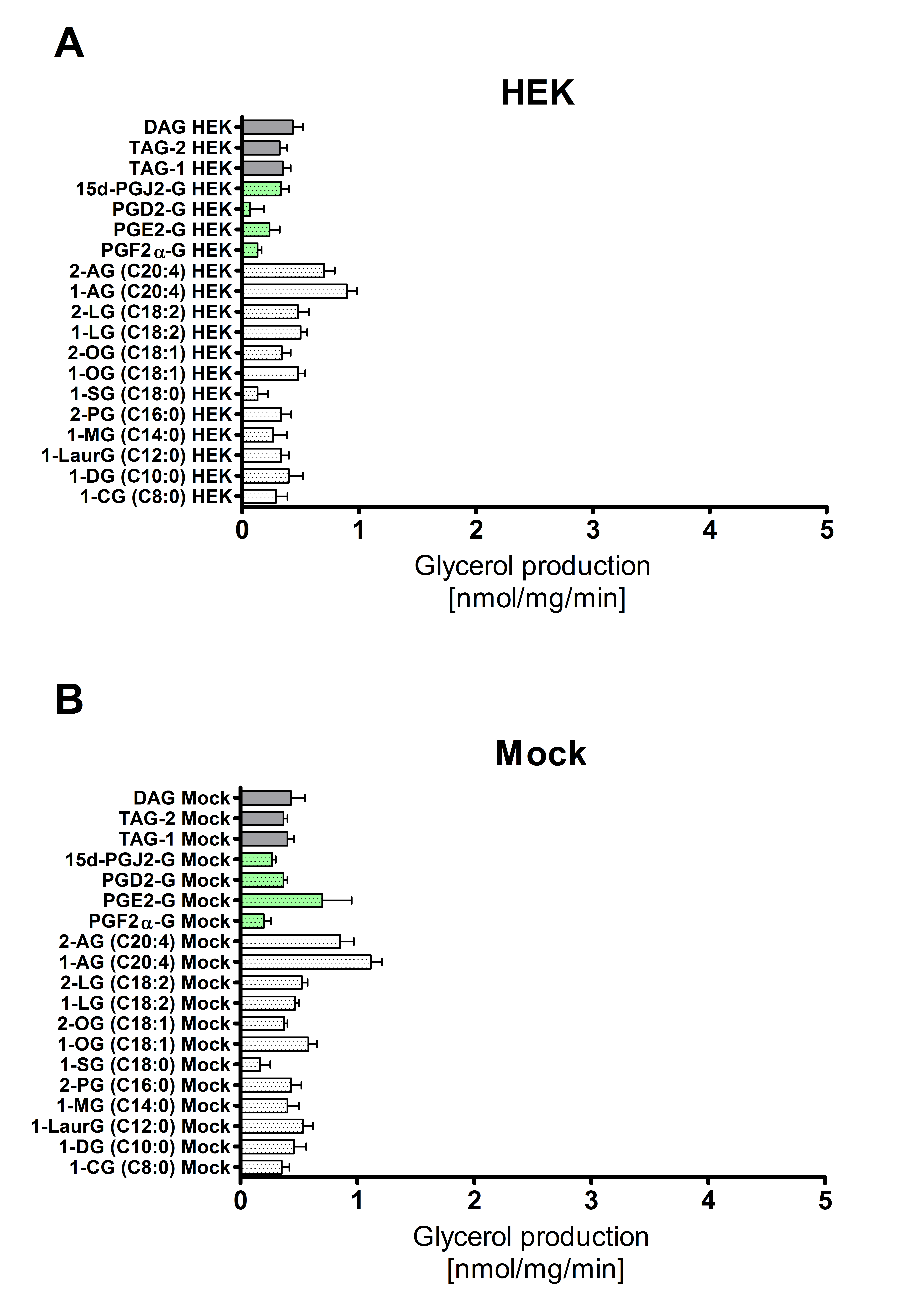

Supplement: Figure S1 — Background activity in HEK293 (A) and Mock-transfected cells (B) towards the 19 substrates tested in this study. The substrate panel included monoacylglycerols (MAGs) with the indicated acyl chain length, isomer and degree of saturation, the diacylglycerol (DAG) 1,2-dioleoyl(C18∶1)-rac-glycerol, the triacylglycerols (TAG-1 = 1,2,3-trioleoyl(C18∶1)glycerol; TAG-2 = 1-palmitoyl(C16∶0)-2-oleoyl(C18∶1)-3-linoleoyl(C18∶2)-rac-glycerol, as well as the prostaglandin glycerol esters PGD2-G, PGE2-G, PGF2α-G and 15d-PGJ2-G. Cellular lysates (0.3 µg/well) were incubated together with the indicated substrates [25 µM final concentration, added from 10 mM stock solutions in ethanol into the glycerol assay mix containing 0.5% (w/v) fatty acid free BSA and 1% (v/v ethanol). Glycerol production was determined at time-point 60 min. Statistical comparisons (unpaired t-test) indicated no significant differences (p>0.05) in the activity between HEK and Mock cells towards any tested substrate. (TIF) [file pone.0109869.s001.tif]

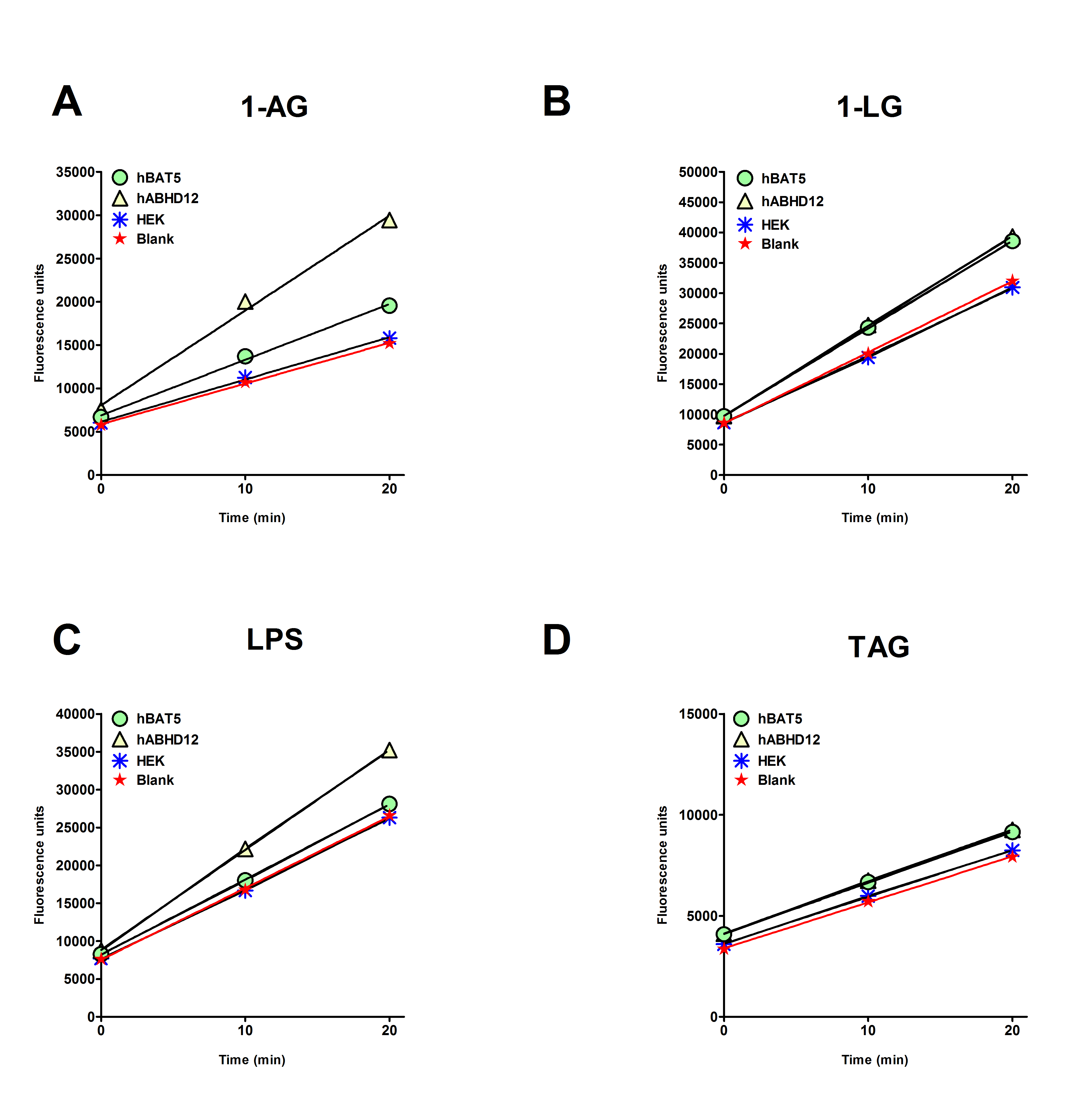

Supplement: Figure S2 — hBAT5-catalyzed hydrolysis of LPS or TAG does not exceed cellular background activity. Shown is time-dependency of fluorescence resulting from fatty acid liberation in incubations of the indicated substrates together with lysates of HEK293 cells with or without overexpression of hBAT5 or hABHD12. HEK293 cells were transiently transfected with the cDNA encoding hBAT5 or hABHD12, as detailed in [10] and the Methods section. After 48 h, cells were harvested and lysates prepared for lipase activity measurements based on the Cayman’s FFA fluorescence assay kit. The substrates included the MAGs 1-AG (A) and 1-LG (B), the lysophospholipid C18∶1-LPS (C) and the TAG 1,2,3-trioleoyl(C18∶1)glycerol (D). Cellular lysates (0.3 µg/well) were incubated together with the indicated substrates [50 µM final concentration, added from 10 mM stock solutions in ethanol into the FFA assay mix containing 0.1% (v/v) ethanol. Each condition included also assay blanks lacking the lysate. Raw fluorescence readings are shown for time-points 0, 10 and 20 min. Data are mean ± SD from duplicate wells. Note absence of hBAT5-catalyzed hydrolysis of LPS and TAG, as evidenced by parallel lines representing Blank, HEK and hBAT5-HEK lysates. Note also that 1-AG and 1-LG are hydrolyzed by hBAT5 and hABHD12 at rates clearly exceeding the cellular background and that LPS is hydrolyzed by hABHD12. (TIF) [file pone.0109869.s002.tif]

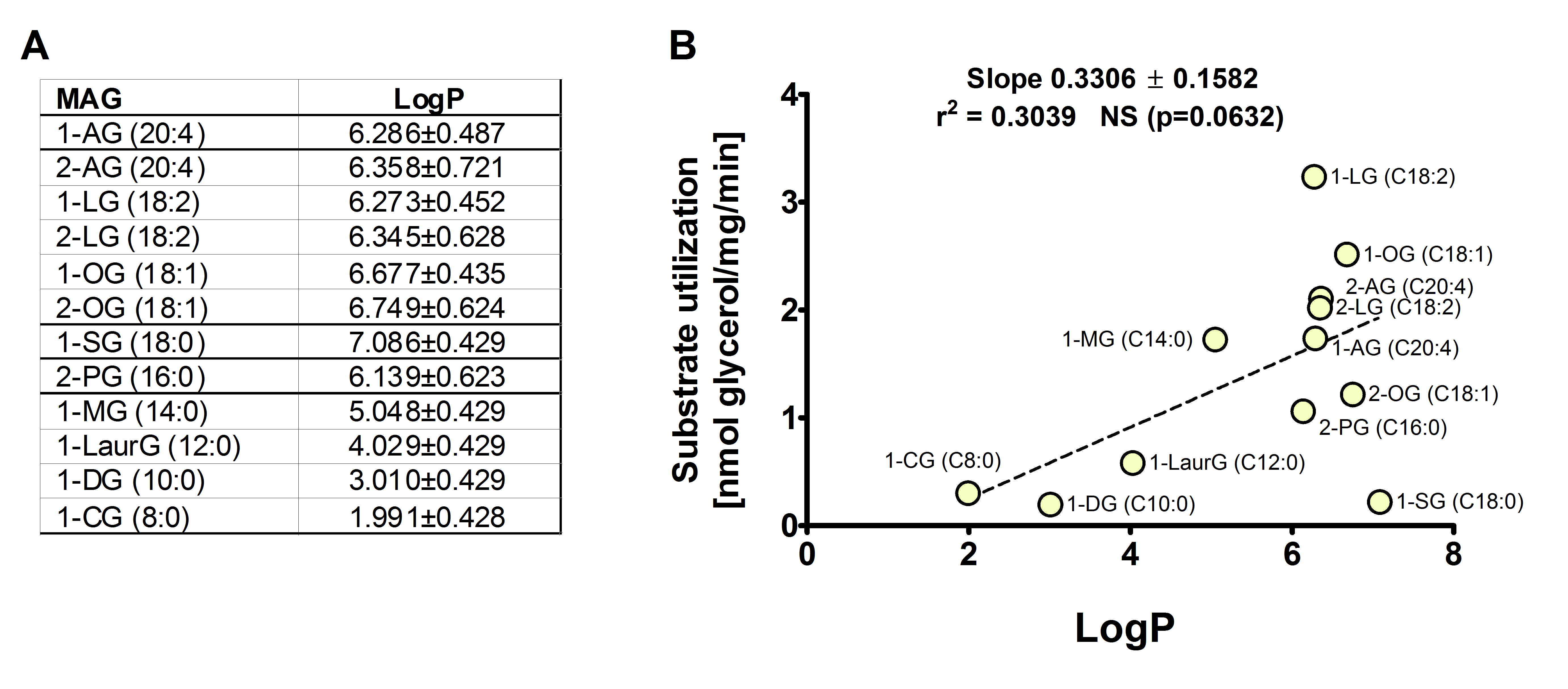

Supplement: Figure S3 — Lipophilicity vs. hBAT5 utilization of the twelve MAG species tested in this study using routine assay conditions with 0.5% (w/v) BSA included. A. LogP values were calculated using Advanced Chemistry Development (ACD/Labs) Software V11.02 (© 1994–2014 ACD/Labs). B. Mean substrate utilization (data extracted from Fig. 3B) plotted against the LogP values. The slope of the best-fit line (dashed, obtained by linear regression analysis) does significantly deviate from zero. (TIF) [file pone.0109869.s003.tif]
